# Transcriptome Architecture of Osteoblastic Cells Infected With *Staphylococcus aureus* Reveals Strong Inflammatory Responses and Signatures of Metabolic and Epigenetic Dysregulation

**DOI:** 10.3389/fcimb.2022.854242

**Published:** 2022-04-07

**Authors:** Aurélie Nicolas, Martine Deplanche, Pierre-Henri Commere, Alan Diot, Clemence Genthon, Wanderson Marques da Silva, Vasco Azevedo, Pierre Germon, Hélène Jamme, Eric Guédon, Yves Le Loir, Fréderic Laurent, Hélène Bierne, Nadia Berkova

**Affiliations:** ^1^ Institut National de Recherche pour l’agriculture, l’alimentation et l’environnement (INRAE), Institut Agro, Science et Technologie du Lait et de l’OEuf (STLO), Rennes, France; ^2^ Cytometry and Biomarkers Centre de Ressources et Recherches Technologiques (C2RT), Institut Pasteur, Paris, France; ^3^ Centre International de Recherche en Infectiologie, CIRI, Inserm U1111, Centre National de la Recherche Scientifique (CNRS) Unité Mixte de Recherche 5308 (UMR5308), Ecole Normale Supérieure (ENS) de Lyon, Universit´ Claude Bernard Lyon 1 (UCBL1), Lyon, France; ^4^ Hospices Civils de Lyon, French National Reference Centre for Staphylococci, Lyon, France; ^5^ Institut National de Recherche pour l’agriculture, l’alimentation et l’environnement (INRAE), Unité Service 1426 (US1426), Transcriptome Plateforme Technologique (GeT-PlaGe), Genotoul, Castanet-Tolosan, France; ^6^ Instituto de Ciências Biológicas, Universidade Federal de Minas Gerais (UFMG), Belo Horizonte, Brazil; ^7^ Institut National de Recherche pour l’agriculture, l’alimentation et l’environnement (INRAE), Université François Rabelais, Infectiologie et Santé Publique (ISP), Tours, France; ^8^ Université Paris-Saclay, Université de Versailles Saint-Quentin-en-Yvelines (UVSQ), Institut National de Recherche pour l’agriculture, l’alimentation et l’environnement (INRAE), Biologie de la Reproduction, Environnement, Epigénétique et Développement (BREED), Jouy-en-Josas, France; ^9^ Ecole Nationale Vétérinaire d’Alfort, Biologie de la Reproduction, Environnement, Epigénétique et Développement (BREED), Maisons-Alfort, France; ^10^ Université Paris-Saclay, Institut National de Recherche pour l’agriculture, l’alimentation et l’environnement (INRAE), AgroParisTech, Micalis Institute, Jouy-en-Josas, France

**Keywords:** *Staphylococcus aureus*, osteoblasts, persistence, transcriptomics, epigenetics, metabolism, immune response

## Abstract

*Staphylococcus aureus* is an opportunistic pathogen that causes a range of devastating diseases including chronic osteomyelitis, which partially relies on the internalization and persistence of *S. aureus* in osteoblasts. The identification of the mechanisms of the osteoblast response to intracellular *S. aureus* is thus crucial to improve the knowledge of this infectious pathology. Since the signal from specifically infected bacteria-bearing cells is diluted and the results are confounded by bystander effects of uninfected cells, we developed a novel model of long-term infection. Using a flow cytometric approach we isolated only *S. aureus*-bearing cells from mixed populations that allows to identify signals specific to intracellular infection. Here we present an in-depth analysis of the effect of long-term *S. aureus* infection on the transcriptional program of human osteoblast-like cells. After RNA-seq and KEGG and Reactome pathway enrichment analysis, the remodeled transcriptomic profile of infected cells revealed exacerbated immune and inflammatory responses, as well as metabolic dysregulations that likely influence the intracellular life of bacteria. Numerous genes encoding epigenetic regulators were downregulated. The later included genes coding for components of chromatin-repressive complexes (*e.g.*, NuRD, BAHD1 and PRC1) and epifactors involved in DNA methylation. Sets of genes encoding proteins of cell adhesion or neurotransmission were also deregulated. Our results suggest that intracellular *S. aureus* infection has a long-term impact on the genome and epigenome of host cells, which may exert patho-physiological dysfunctions additionally to the defense response during the infection process. Overall, these results not only improve our conceptual understanding of biological processes involved in the long-term *S. aureus* infections of osteoblast-like cells, but also provide an atlas of deregulated host genes and biological pathways and identify novel markers and potential candidates for prophylactic and therapeutic approaches.

## Introduction

The Gram-positive bacterium *Staphylococcus aureus* (*S. aureus*) is an opportunistic pathogen that causes a panel of diseases ranging from mild skin infections to life-threatening infections, such as septicemia, endocarditis, pneumonia or bone joint infection (BJI) including osteomyelitis (Kalinka et al., 2014). Originally considered as an extracellular pathogen, *S. aureus* has been detected inside of osteoblasts, where it is likely involved in the development of chronic osteomyelitis *via* the formation of small colony variant (SCV) phenotypes (Tuchscherr et al., 2016). A timely diagnosis and the understanding of the molecular pathophysiology are pivotal to improve the outcomes of osteomyelitis. However, biomarkers and specific pathways are difficult to identify with current clinical expertise. The development of modern methods for gene expression analysis allowed identifying genes and pathways that are involved in various *S. aureus*-associated infection, such as osteoarticular infection, which demonstrated over-expression of genes involved in the coagulation cascade and platelet adhesion (Banchereau et al., 2012) or an activation of immune system genes and a repression of metabolic genes in a *S. aureus* skin infection (Brady et al., 2015). However, the whole picture of the host factors and related pathways, as well as a comprehensive insight of the relationship between different processes induced by internalized bacteria through dissecting the layers of gene regulation, is still missing.

In an invasive bacterial process, a succession of phenomena occur, such as bacterial adhesion, internalization, survival and intracellular persistence of the pathogen, or clearance of the infection with eventual death of infected host cells (Rollin et al., 2017; Peyrusson et al., 2020). During long-term infection with *S. aureus*, only a small subpopulation of cells carries intracellular bacteria (Tuchscherr et al., 2011), however these cells likely influence the outcome of the infection. The overall transcriptional response of the infected host/tissue is the average between the response of infected cells and that of uninfected cells (Chattopadhyay et al., 2018). Therefore, the signal of uninfected cells dilutes the signal generated by the infected cells and thus weakens the information on the host-pathogen relationship during intracellular infections. The detection of some key participants of the interaction may thus become impossible due to the dilution of their signal below the limit of detection, whereas a non-specific signal from dominant uninfected bystander cells overpasses the infected-cells specific signal (Chattopadhyay et al., 2018). New approaches that can distinguish the infected cell response from that of uninfected cells are therefore required in order to better understand the mechanisms of the *S. aureus*-host interactions.

In the current work, we described the development of a new model of *S. aureus* infection of human osteoblast-like cells. Since the signal from specifically infected bacteria-bearing cells is diluted and the results are confounded by bystander effects of uninfected cells we isolated only *S. aureus*-bearing cells from mixed populations using a flow cytometric approach. An employment of RNA-seq methodology, which allows in-depth transcriptome analysis (Saliba et al., 2014), has enabled transcriptomic profiling of host genes at an unprecedented scale and resolution. In particular, we present the identification of understudied host genes and pathways, such as certain metabolic and epigenetic pathways, in addition to conventional defense genes. This improves our conceptual understanding of the biological processes involved in the development of *S. aureus* infections, with a focus on chronic osteomyelitis, and allows us to propose the use of a network of new biomarkers and also highlights potential candidates for the development of new prophylactic and therapeutic approaches.

## Material and Methods

### Maintenance of Eukaryotic Cells

The human osteoblast-like MG-63 cell line (LGC Standards, Teddington, UK) is derived from a juxtacortical osteosarcoma diagnosed in the distal diaphysis of the left femur of a 14-year-old male (Billiau et al., 1977). MG-63 cells were cultured in cDMEM (DMEM, GlutaMax, 10% fetal calf serum (Gibco) supplemented with 100 U/mL penicillin, and 100 μg/mL streptomycin) at 37°C in 5% CO_2_. Trypsin/EDTA (Sigma) was used for cells subculturing.

### *Staphylococcus aureus* Strains and Culture Conditions

We used the following *S. aureus* strains: SA113, which is derived from NCTC 8325 strain isolated from a conjunctiva of a patient with corneal ulcer, and mCherry*SA113* (pctuf-mCherry) strain, which bears a vector expressing mCherry marker (red fluorescence) fused to the propeptide of lipase for fluorescence enhancement (a kind gift from Pr. Friedrich Götz, Laboratory of Microbial Genetics, University of Tübingen, Germany) (Mauthe et al., 2012). *S. aureus* cultures were performed as described (Deplanche et al., 2015). Aliquots from overnight cultures on Brain Heart Infusion (BHI) broth were diluted (1:50) in DMEM. Strains were grown in 50 mL tubes and incubated at 37°C under anaerobic conditions until cultures had reached an optical density of 0.6 at 600 nm, corresponding to approximately 10^8^ CFU/mL (CFU, colony-forming unit). The staphylococci were harvested by centrifugation, washed twice with phosphate-buffered saline (PBS), and resuspended in the interaction medium (DMEM). Bacterial concentrations were estimated spectrophotometrically and the number of live bacterial cells was confirmed by plate counts.

### Development of the *S. aureus* Infection Model

In the course of a long-term *S. aureus* infection there is a small population bacteria-bearing cells that likely influences the outcome of the infection. Therefore, to isolate the population of cells containing internalized *S. aureus* we established a flow cytometry-based assay using the selection of only host cells bearing mCherry*-*expressing *S*. *aureus SA113*. MG-63 cells were grown in 75 ml flasks. We optimized host cell growth and multiplicity of infection (MOI), in order to limit the cytotoxicity of infection (Alekseeva et al., 2013), and achieved the best results with 60% of host cell confluence and a MOI of 25 bacteria per cell at the onset of infection. Bacterial concentrations were estimated spectrophotometrically and were confirmed by determination of CFU. Extracellular bacteria were removed 2 h post-infection by incubating cells in cDMEM with 20 μg/mL lysostaphin and 100 μg/mL gentamicin for 2 h, which eliminates extracellular bacteria without altering intracellular bacteria (Deplanche et al., 2019; Lima Leite et al., 2020) followed by incubation in cDMEM containing 25 μg/mL of gentamicin and 3% of FCS. The low concentration of FCS was used in order to slow down a cell proliferation rate during a long-term infection. After 3 days the incubation medium was replaced with the fresh medium containing 25 μg/mL of gentamicin with 3% of FCS removing cell debris and cells were incubated for additional 3 days. The cell death was estimated by the release of LDH (Pierce LDH Cytotoxicity Assay Kit; Pierce, Rockford, IL, USA) according to manufacturer’ instructions, as we described previously (Deplanche et al., 2015). Then, cells were trypsinized, collected, centrifuged, and prepared either for RNA-seq, fluorescence microscopy or cytofluorometry analysis. The determination of the amount of internalized bacteria was carried as previously described (Alekseeva et al., 2013; Bouchard et al., 2013). Briefly, following 2 h of infection, infected cells were lysed with 0.05% Triton X-100 in PBS, and cell lysates were plated on BHI agar at different dilutions. CFU were determined after overnight incubation.

Different infection times (from 1 to 9 days) have been tested to find the longest incubation time to model a long-term, persistent infection and to extract, after Fluorescence-activated Cell Sorting (FACS), a quantity of host RNA sufficient for RNA sequencing experiments. Infected and uninfected control cells were trypsinized and sorted at a rate of approximately 8,000 events/sec using MoFLO Astrios fluorescent cell sorter. The sorting was carried out on a MoFLO Astrios “Beckman Coulter” sorter with 488- and 561-nm lasers at 200 mW. The sorting was carried out, with a 100 μm nozzle at a pressure of 25 PSI and a differential pressure with the sample of 0.3 to 0.4 PSI. The sheath liquid NaCl 0.9% (Revol Company) was filtered on a 0.04-μm filter. The mCherry fluorescence was detected with a 614/20 filter and excited with the 561 nm laser.

### Fluorescence Microscopy

Cells that have been sorted by FACS were placed onto the slides. Afterwards, cells were fixed with 4% paraformaldehyde (PFA) in PBS for 20 min. The cover slips were then mounted on slides with DAPI-containing ProLong antifade Vectashield medium (Vector Laboratory, Les Ulis, France). Specimens were imaged with a Zeiss fluorescence microscope using ×400 magnification.

### Gene Expression Analysis by Real-Time Quantitative Reverse Transcription PCR (RT-qPCR)

The infection of human MG-63 osteoblast-like cells was performed as indicated above. The expression of selected genes of infected MG-63 cells was evaluated by quantitative real-time PCR (RT-qPCR), as described previously (Deplanche et al., 2015). Briefly, total RNA was isolated from MG-63 cells with an RNA II kit (Macherey-Nagel). RNA concentration and purity were assessed using a Nanodrop spectrophotometer (Thermo Scientific). A cDNAs were synthesized using a qScript cDNA synthesis kit (Quanta Biosciences). Reaction mixtures devoid of reverse transcriptase and reaction mixtures containing H_2_O instead of cDNA were used as negative controls. Each reaction was performed in triplicate. Primer sequences were designed using Primer 3. The list of primers is presented at **Supplementary Data, Table 1**. *PPIA*, *GAPDH*, *PGK1*, *HRPT1*, *TBP* and *HSP90AB1* were used as normalizer genes. Amplification was carried out on a CFX96 Real Time System (Bio-Rad) for 3 minutes at 95°C and 40 cycles of 2 steps consisting of 5 seconds at 95°C and 30 seconds at 60°C. The relative quantification of the mRNA levels of the target genes was determined using CFX Manager based on the ΔΔCT-method (Livak and Schmittgen, 2001). The six genes from RNAseq data were selected as potential normalizer genes according to their most stable expression. The expression stability of those genes was confirmed by using of the Gene Expression Module of CFX Manager (Bio-Rad). The amount of target was normalized to normalizer (housekeeping) genes. Relative quantification after normalization refers to the PCR signal of the target transcript in a treatment group divided by the values obtained from uninfected control cells, arbitrarily set to 1. When the expression was decreased compared to that in uninfected control cells, data were presented as negative values.

### RNA Sequencing

Three biological replicates of uninfected control cells and specifically infected *S. aureus*-bearing cells that were isolated by a flow cytometric approach from infected mixed cell populations containing cells with and without internalized bacteria, were used for the RNA sequencing and analysis.

Total RNA from each sample was isolated with an RNA II kit (Macherey-Nagel) according to manufacturer’s instructions with a subsequent DNase treatment (Dnase Rnase free, Ambion) according to the supplier. RNA concentrations were quantified using a Nanodrop. RNA quality (RIN) was evaluated using an Agilent 2100 bioanalyzer (Agilent Technologies, Santa Clara, CA). RNA labeling and hybridization were performed at the GeT-PlaGe core facility, INRAE Toulouse, France. All of the RNA samples had a RIN value greater than 8.2, indicating a good RNA integrity. The ratio 260/280 were greater than 2 indicating a good RNA quality (**Supplementary Data, Table 2**). RNA-seq libraries have been prepared according to Illumina’s protocols using the Illumina TruSeq Stranded mRNA sample prep kit to analyze mRNA. Briefly, mRNAs were selected using poly-T beads. Then, RNAs were fragmented to generate double stranded cDNA and adapters were ligated to be sequenced. Eleven cycles of PCR were applied to amplify libraries. Library quality was assessed using a Fragment Analyser and libraries were quantified by qPCR using the Kapa Library Quantification Kit. RNA-seq experiments were performed on an Illumina HiSeq3000 using a paired-end read length of 2x150 pb with the Illumina HiSeq3000 sequencing kits. Adapters were removed with Trim galore (v 0.4.0) (Martin, 2011) and data quality was assessed using FastQC (v 0.11.2), both from the Babraham Institute.

### RNA-Seq Analysis

Reads were quality trimmed with Sickle (v 1.210) in “pe” (pair-end) mode with default parameters. Paired sequences were then mapped to human reference genome (GhCR38.80) with Tophat (v 2.0.14) (Trapnell et al., 2009) with default parameters. Genes were counted with htseq-count (v 0.6.1) (Anders et al., 2015). DeSEQ2 (Love et al., 2014), an R package embedded in the package SARTools (v 1.2.0) (Varet et al., 2016) was used to normalized the count table with 29,195 genomic features expressed and generate a list of differentially expressed genes (DEGs). A Benjamini-Hochberg p-value adjustment, a multiple testing correction, is performed to control the false positive rate. The threshold of statistical significance is set to 0.05. Positive and control samples ranged from 41 to 85 million pair-end reads per sample and 51 to 94 million pair-end reads per sample, respectively (**Supplementary Data, Table 2**).

Principal Component Analysis indicated that the biological variability (positive infected *vs* control uninfected samples) was the main source of data variance (**Supplementary Data, Figure 1**). Lists of DEGs were annotated with Biomart (Smedley et al., 2015) from Ensembl (Cunningham et al., 2015) with the version GRCh38p5.

### Functional Annotation

Gene-set enrichment tests in Kyoto Encyclopedia of Genes and Genomes (KEGG) pathways were performed with the R packages, GAGE (Luo et al., 2009). Gene sets with adjusted p-value < 0.05 were considered as being significantly enriched. A network of KEGG DEGs set was constructed using R package, FGnet (Aibar et al., 2015). A final network diagram was drawn with Cytoscape (Shannon et al., 2003). Gene-set enrichment tests in Reactome pathways (Jassal et al., 2020) were performed with R package ReactomePA (Yu and He, 2016) with gsePathway function. Gene sets with adjusted *p*-value < 0.05 were considered as being significantly enriched. We also used the Epifactor and DAVID databases (Huang et al., 2009; Medvedeva et al., 2015).The datasets presented in this study can be found in online repositories. The repository and accession numbers can be found at: https://www.ebi.ac.uk/ena/browser/view/PRJEB47070.

### Enzyme-Linked Immunosorbent Assay (ELISA)

Cell culture supernatants were subjected to detection of periostin and cytokines by sandwich-ELISA (RD system) according to the manufacturer’s instructions. Briefly, wells of 96-well plates were coated with capture antibody and were incubated overnight. All incubations were done at room temperature. After washing with the wash buffer (PBS + 0.05% Tween 20), the wells were incubated with the reagent dilution buffer for 1h. Then tested samples were added to the appropriate wells. After 2h of incubation, biotin-conjugated detection antibody was added to the wells for 2h. Then, Streptavidin-HRP solution was added and incubated for 20 min in the dark. The reaction was stopped with stop solution, and absorbance was read at 450 nm.

### Statistical Analysis

Three biological replicates in triplicates were performed for ELISA experiments. Results were pooled from 3 biological replicates with each being an average of triplicates. The differences among the groups were assessed by ANOVA. P<0.05 was considered significant. Tukey’s honestly significant difference test was applied for comparison of means between groups. The values are expressed as mean ± SD.

## Results

### Development of an *S. aureus* Infection Model in Osteoblast-Like Cells

**Figure 1:**
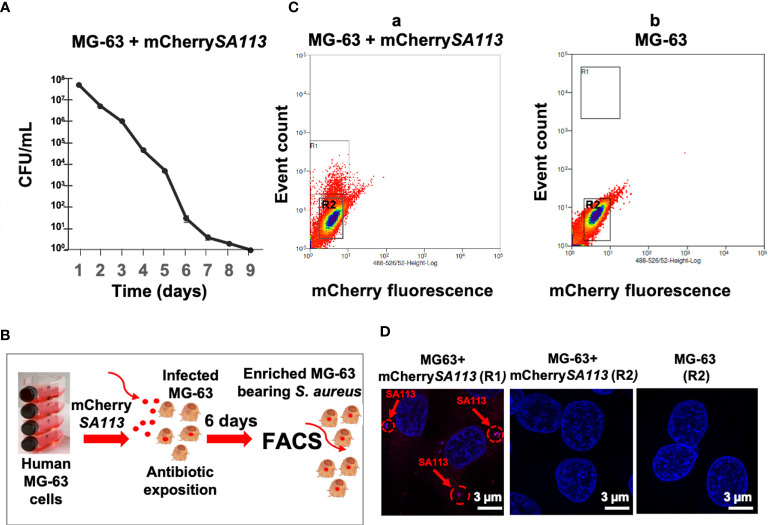
**(b)** Development of the infection model to isolate solely cells containing internalized *S. aureus.* **(A)** Survival curve of mCherry*SA113* bacteria internalized into MG-63 cells (CFU counts). **(B)** Scheme of the FACS-based purification of mCherrySA113-containing MG-63 cells. Extracellular bacteria were removed 2 h post-infection by incubating cells in cDMEM with 20 μg/mL lysostaphin and 100 μg/mL gentamicin for 2 h, which eliminates extracellular bacteria followed by incubation in cDMEM containing 25 μg/mL of gentamicin. **(C)** *S. aureus* infected **(a)** and uninfected control **(b)** MG-63 cells were trypsinized and sorted at a rate of 8,000 events/sec using MoFLO Astrios Beckman Coulter fluorescent cell sorter. The R1 and R2 region **(a)** correspond to sorted cells either containing mCherrySA113 bacteria (R1) or without bacteria in the infected cell culture (R2). The R2 region **(b)** corresponds to sorted non-infected cells. **(D)** Representative fluorescence microscopy images of sorted cells, from R1, R2 **(C, a)** or R2 **(C, b)** FACS samples, imaged with a Zeiss fluorescence microscope using ×400 magnification. Nuclei were stained with DAPI (blue staining). Red arrows and dotted circles indicate internalized mCherrySA113 bacteria (red staining). Scale bar: 3 μm.

To characterize the *S. aureus*-host relationship in a physiological setting corresponding to a long-term infection of non-immune cells, we developed a model of osteoblast-like cells bearing internalized *S. aureus* using conditions described in Material and Methods. First, we determined the optimal duration of *S. aureus* incubation with osteoblasts, by quantifying the intracellular loads of *S. aureus* from day 1 to day 9 (d1 to d9) post-infection (p.i.). As shown in **Figure 1A**, the number of internalized bacteria (assessed by CFU counts) progressively decreased from the onset of infection until a dramatic decrease on d7 p.i., likely due to the activation of the host bacterial clearance system. Thereafter, we choose to use d6 p.i., as this time point mimics a long-term intracellular infection stage of *S. aureus*, while allowing the isolation of sufficient number of infected cells to extract the quantities of host RNA required for RNA-seq (**Figure 1B**). At d6 p.i., cells reached 96±2% of the confluence and the cell viability was 95±2% as estimated by the release of LDH (Pierce LDH Cytotoxicity Assay Kit). Host cell fluorescence detectable by flow cytometry was associated with bacterial internalization, as confirmed by fluorescence microscopy (**Figures 1C** **, D**). With an MOI of 25 at the onset of infection, 5% of MG-63 osteoblasts exhibited mCherry fluorescence (mCherry+) at d6 p.i. (based on a gate drawn at the 99^th^ percentile of fluorescence in uninfected control cells) (**Figure 1C**). Analysis of FACS-sorted cells by fluorescence microscopy confirmed that the fluorescence of most cells (**Figure 1C**, **a**; R1 region) was indeed attributable to internalized bacteria (**Figure 1D**). In addition, the central region of the dot plot (**Figure 1** **C, a, b**; R2 region) was composed of cells lacking detectable intracellular bacteria (**Figure 1D**).

### Transcriptional Profiling of *S. aureus-*Infected MG-63 Cells

To identify the transcriptional changes induced by a 6-day infection in a sorted population of infected cells, mRNA of osteoblast-like cells either hosting intracellular *S. aureus* (positive) or remaining non-infected (control) were analyzed by RNA-seq, with three samples by condition. Transcriptomic analysis was performed on 29,195 human genomic features, highlighting 2,850 differential expressed genes (DEGs), defined as protein-coding genes that were statistically differentially expressed in *S. aureus*-infected cells compared to uninfected controls (adj. *p*-value <0.05), with a threshold log2 fold change (FC) -0.3 > log_2_FC > 0.3. 1,514 of those DEGs were upregulated, while 1,336 were downregulated (**Table S1**). 401 genes were at least three-fold enriched (log_2_FC ≥ 1.5), while 208 genes were three-fold less abundant (log_2_FC ≤ -1.5) in infected cells. Among them, 153 genes were eight-fold enriched (log_2_FC ≥ 3) and 41 genes were eight-fold less abundant (log_2_FC >-3 (**Table S1**).

To validate RNA-seq gene expression profiles, the expression of 30 genes involved in different processes was assessed by RT-qPCR. Results were in agreement with RNA-seq data (**Table 1**).

**Table 1:** Validation by Quantitative real-time–PCR (qPCR) analysis of various significantly differentially expressed genes from the RNA-seq dataset.

UniProt ID	Gene name	Gene description	Fold Change RT-qPCR (SE)	Fold ChangeRNA-seq
		**METABOLISM**		
O60218	*akr1b10*	aldo-keto reductase family 1 member B10	3.83 (0.22)	117.85
O95992	*ch25h*	cholesterol 25-hydroxylase	3.74 (0.23)	6.26
P00167	*cyb5a*	cytochrome b5 type A (microsomal)	4.37 (0.21)	24.61
Q8TDS4	*hcar2*	hydroxycarboxylic acid receptor 2	4.37 (0.31)	13.22
Q9Y5L2	*hilpda*	hypoxia inducible lipid droplet-associated	2.93 (0.12)	3.16
P28845	*hsd11b1*	hydroxysteroid (11-beta) dehydrogenase 1	3.54 (0.17)	53.22
P43490	*nampt*	nicotinamide phosphoribosyltransferase	4.15 (0.19)	5.51
Q6PCE3	*pgm2l1*	phosphoglucomutase 2-like 1	3.68 (0.27)	4.37
PDJI9	*saa2*	serum amyloid A2	4.38 (0.26)	48.93
Q9H2J7	*slc6a15*	solute carrier family 6 (neutral amino acid transporter) member 15	3.21 (0.18)	4.56
P04179	*sod2*	superoxide dismutase, mitochondrial	4.22 (0.24)	14.24
O95497	*vnn1*	vanin 1	7.84 (0.17)	12.24
		**DEFENSE**		
P04003	*c4bpa*	complement component 4 binding protein, alpha	6.10 (0.22)	26.61
P1583	*il1a*	interleukin 1 alpha	6.93 (0.21)	14.75
O14508	*socs2*	suppressor of cytokine signaling 2	2.35 (0.09)	5.57
P04141	*csf2*	colony stimulating factor 2 (granulocyte-macrophage)	30.52 (1.92)	1316.82
P80162	*cxcl6*	chemokine (C-X-C motif) ligand 6	52.39 (1.97)	174.86
P01584	*il1b*	interleukin 1 beta	7.08 (0.21)	40.50
P24001	*il32*	interleukin 32	8. 69 (0.25)	16.83
		**CELL JUNCTIONS**		
P35609	*actn2*	actinin, alpha 2	2.17 (0.11)	2.88
P55289	*cdh12*	cadherin 12, type 2 (N-cadherin 2)	3.01 (0.13)	4.63
P33151	*cdh5*	cadherin 5, type 2 (vascular endothelium)	0.25 (0.04)	0.10
O95832	*cldn1*	claudin 1	10.41 (0.23)	22.18
Q15063	*postn*	periostin, osteoblast specific factor	0.35 (0.15)	0.11
		**OTHERS**		
O60437	*ppl*	periplakin	0.24 (0.11)	0.15
Q9H4E5	*rhoj*	ras homolog family member J	0.37 (0.14)	0.18
P17936	*igfbp3*	insulin like growth factor binding protein 3	9.34 (0.34)	12.98
Q12879	*grin2a*	glutamate receptor, ionotropic, N-methyl D-aspartate 2A	3.39 (0.19)	7.10

**Figure 2:**
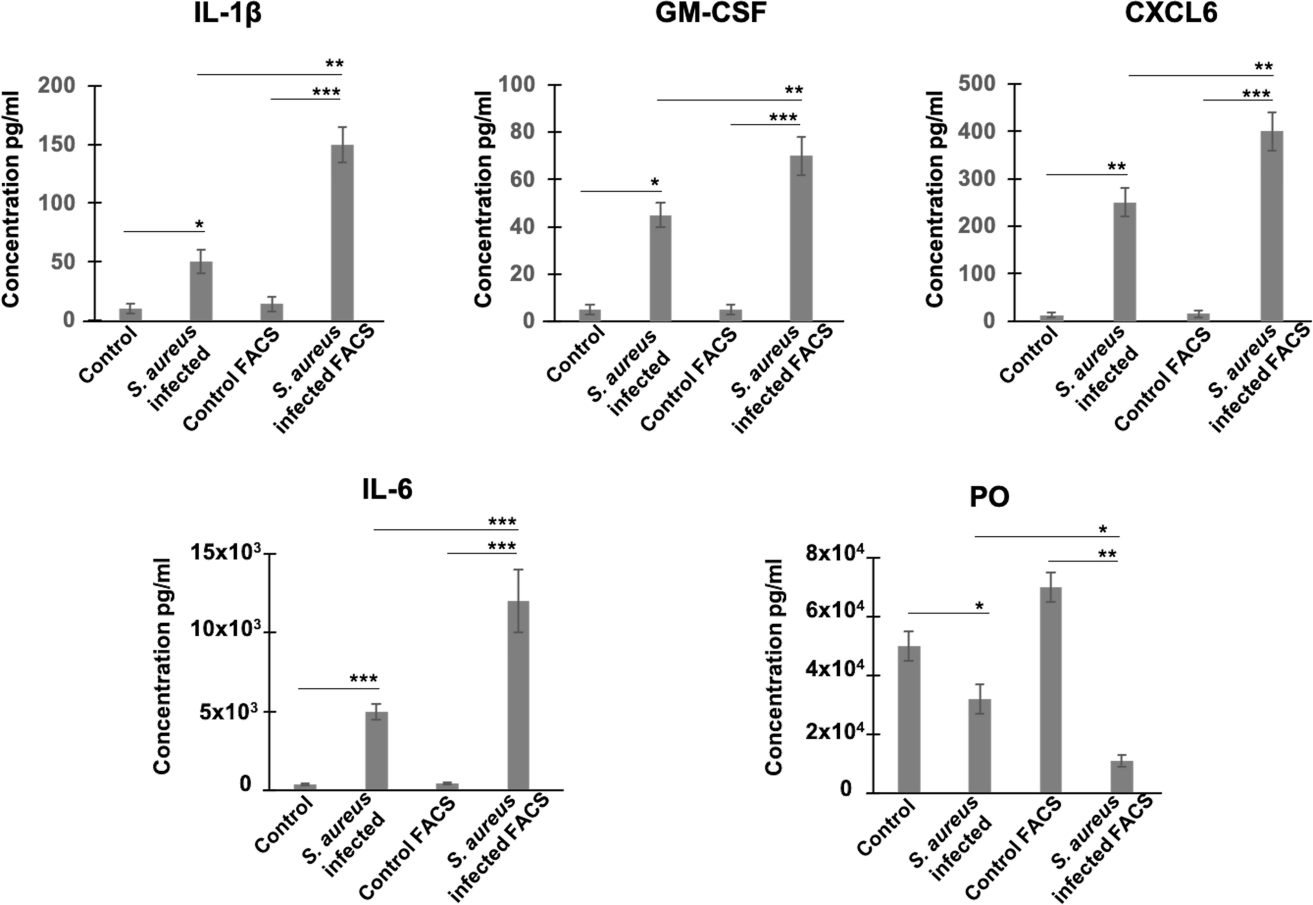
Estimation of protein levels of periostin and inflammatory cytokines in cell supernatants by ELISA. Control and *S. aureus*-infected MG-63 cells at 72h p.i. were trypsinized and either centrifuged to collect supernatants or sorted using MoFLO Astrios fluorescent cell sorter. Afterwards, cells were resuspended in cDMEM and incubated for additional 12 h. Levels of cytokines (IL-1β, GM-CSF, CXCL6, IL-6) and periostin (PO) in two groups of supernatants (1) infected *vs* control cells; (2) sorted by FACS infected cells *vs* sorted by FACS control cells, were assessed by ELISA. Results were pooled from 3 biological replicates with each being an average of 3 experimental replicates. All graphs depict mean ± SD. All data were analyzed using ANOVA following Turkey’s HSD *Post Hoc* test. (*P ≤ 0.05. **P ≤ 0.01. ***P ≤ 0.001).

We then examined whether RNA level alterations corresponded to changes in protein abundance, focusing on several secreted factors. We chose periostin, an osteoblast-specific factor involved in the regulation of cell adhesion and organization of extracellular matrix (Sugiura et al., 1995; Kudo and Kii, 2018), as representative of the product of a down-regulated gene, and as four cytokines/chemokines, CSF-G, CXCL6, IL-1β, and IL-6, as representative products of up-regulated genes. Protein levels in supernatants of heterogeneous infected cell populations (i.e., a mixture of bacteria-bearing and bacteria-free cells), as well as in supernatants of FACS-sorted bacteria-bearing cells were compared to uninfected cells under the same conditions, using ELISA quantification. The results showed that secretions of CSF-G, CXCL6, IL-1β, IL-6 increased, while secretion of periostin decreased, in FACS-sorted and unsorted *S. aureus*-exposed cells compared to control cells (**Figure 2**). These results thus corroborate the RNA-seq data. In addition, and importantly, the sorting procedure amplified the difference in the amount of secreted protein between infected and control cells, compared to unsorted heterogeneous cell populations demonstrating the increased magnitude of infection-induced deregulation.

### Gene-Set Enrichment Analysis (GSEA) Using KEGG Database

**Figure 3:**
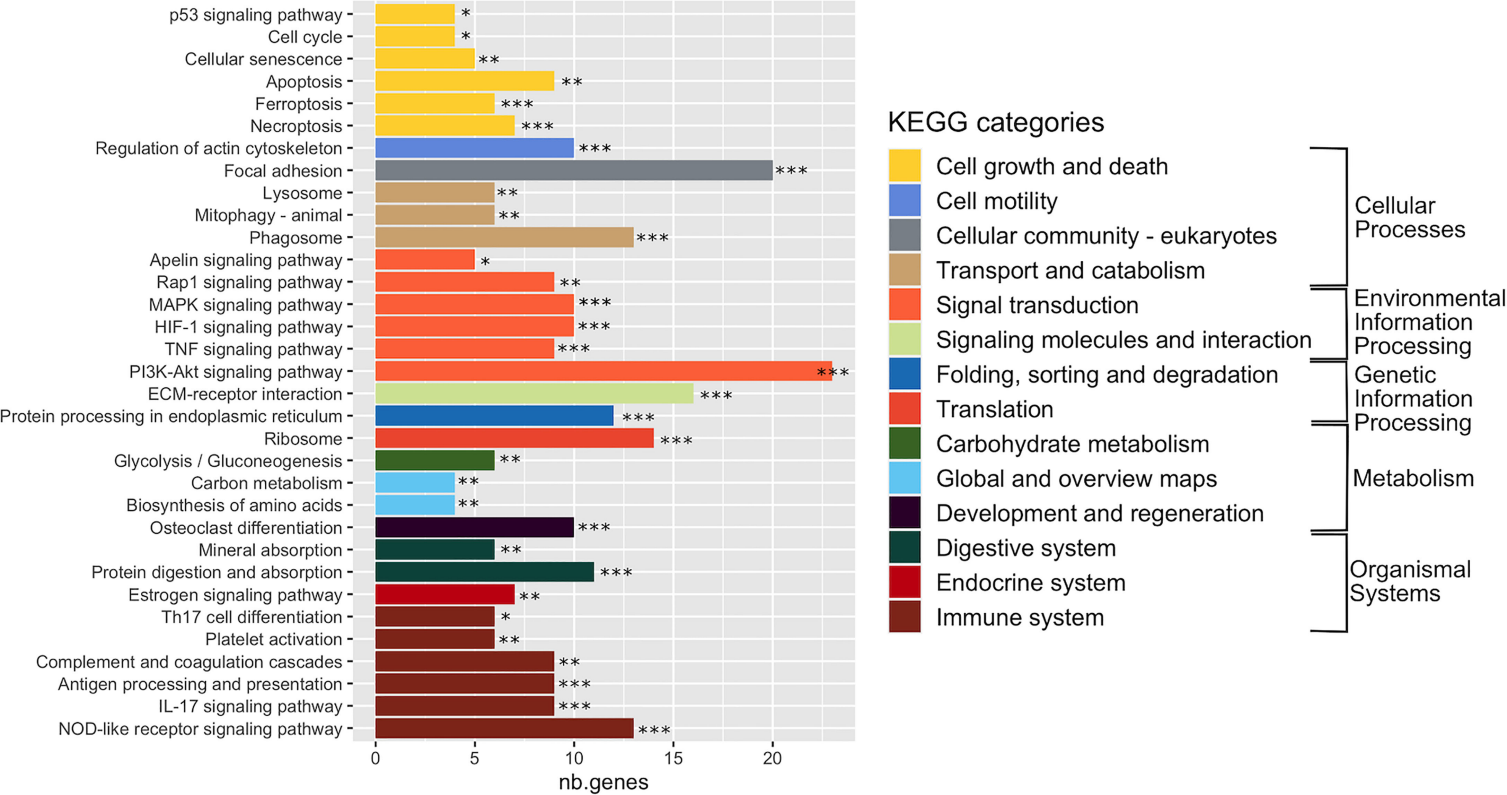
Enriched KEGG pathways in infected cells. Gene-set enrichment analysis was performed in *S. aureus*-bearing cells compared to uninfected control cells by GAGE software with KEGG database. The 33 significant enriched pathways are displayed with number of differentially expressed genes involved. *adjusted p-val < 0.01, ** adjusted p-val < 0.05, *** adjusted p-val < 0.01. Pathways are ordered and colored according to parent category at level 2.

In order to interpret gene expression data in association with biological processes or molecular functions, we performed a GSEA with the GAGE tool on all expressed genes (29,195 genes) (**Table S2**). This analysis revealed 33 significantly enriched KEGG pathways (adj. *p*-val < 0.05) (**Figure 3** and **Table S3**), organized into five functional families: cellular processes (11 pathways), organismal systems (10 pathways), environmental information processing (7 pathways), metabolism (3 pathways), and genetic information processing (2 pathways). More than half of the enriched pathways belonged to functional categories related to signal transduction, immune system and cell growth and death (**Figure 3** and **Table S3**). The signal transduction category included the *PI3K-Akt signaling pathway* (with the highest number of DEGs: 23 DEGs), which negatively mediates NF-κB-dependent inflammation (Lv et al., 2019)*, HIF-1 pathway* that stimulates aerobic glycolysis during *S. aureus* infection (Wickersham et al., 2017), as well as the *MAP kinase*, *TNF, Rip1* and *Apelin* signaling pathways. The immune response category included *antigen processing and presentation, complement and coagulation cascade*, *platelet activation*, *TH17 cell differentiation* and *IL-17 pathways* that are involved in the defense during *S. aureus* infection (Liu et al., 2013a; Fruman et al., 2017), the *NOD-like receptor pathway*, which activates innate and adaptive immune responses (Thammavongsa et al., 2015; Dey and Bishayi, 2017; Narita et al., 2019).

The cell growth and death category, in addition to the *P53 signaling pathway*, included *apoptosis, necroptosis* and *ferroptosis* pathways (Yang and Stockwell, 2016; Jorgensen et al., 2017), as well as the *cell cycle pathway* (that we previously showed to be impacted by *S. aureus* infection) (Deplanche et al., 2015) and the *cellular senescence pathway*, which either promotes a favorable conditions for pathogen survival or acts as an defense mechanisms limiting the rate of infection (Humphreys et al., 2020).

Regarding other enriched pathways, we particularly noticed the transport and catabolism, cell motility, and cellular community KEGG categories, as they could manage the interface of the detection, sequestration and elimination of internalized microbes. The transport and catabolism category mediates activities of organelles that detect cellular signals, followed by the execution of responses during infections. This category encompasses the *phagosome and lysosomes signaling pathways* (Inpanathan and Botelho, 2019), as well as the *mitophagy pathway*, a regulator of NLRP3 inflammasome activation (Kim et al., 2016). The cell motility and cellular community categories included 30 DEGs involved in *focal adhesion* or *actin cytoskeleton* pathways. Another remarkable group of enriched pathways belonged to metabolism, which includes genes belonging to *glycolysis/glyconeogenesis*, *biosynthesis of amino acids* and *carbon metabolism pathways*. Finally, an infection-mediated deregulation of genes belonging to the *estrogen signaling pathway* and the *osteoblast differentiation pathway* was noteworthy (Kovats, 2015; Montecino et al., 2021).

### GSEA Using Reactome Database

**Figure 4:**
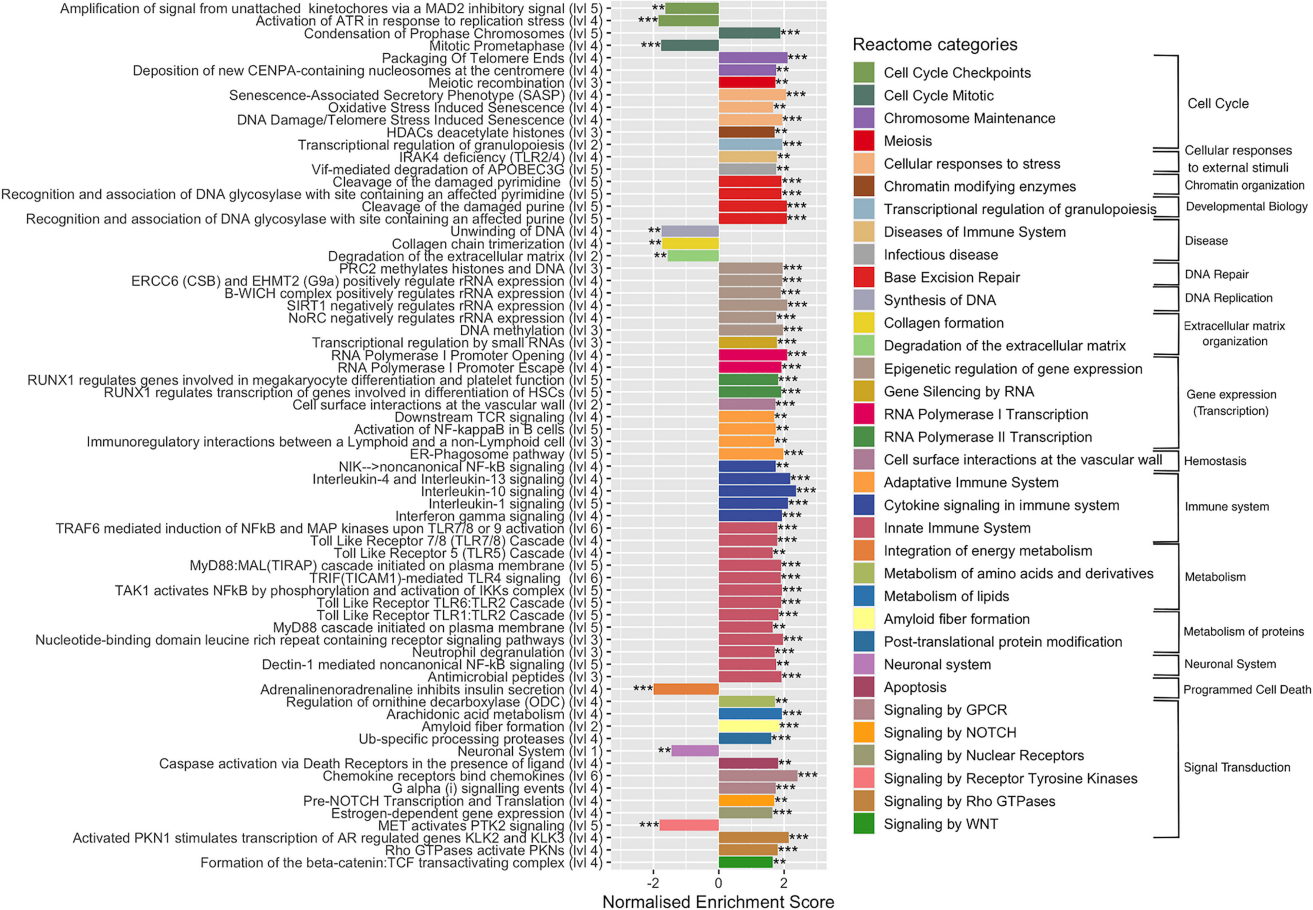
Enriched Reactome pathways in infected cells. Gene-set enrichment analysis was performed in *S. aureus*-bearing cells compared to uninfected control cells by ReactomePA software. The deepest pathways are kept and drawn with the level in hierarchy indicated besides names. Pathways are colored accordingly to parent category at level 2. The normalized enrichment score (NES) is indicated. Negative NES for pathways globally down-regulated and positive NES for pathways globally up-regulated. ** adjusted p-val < 0.05, *** adjusted p-val < 0.01.

To complete functional gene analysis, GSEA was also carried out on the 29,195 expressed genes with the Reactome database, which is more detailed and largest than KEGG database with up to six hierarchical levels (Chowdhury and Sarkar, 2015). The number of Reactome enriched pathways was much higher compared to those identified by KEGG, 106 *vs* 33, respectively. Reactome GSAE overestimates enriched pathways by considering that all pathway levels are enriched at the same time. To facilitate and clarify further analyses, we removed the redundancy of enriched pathways by considering only the deepest. This resulted in 70 Reactome enriched pathways: 61 upregulated and 9 downregulated pathways (**Figure 4** and **Table S4**). This analysis confirmed the deregulation of the pathways identified according to the KEGG database, but with a more precise categorization and a higher number of cellular processes. For instance, Reactome identified 22 upregulated pathways associated with the immune systems, such as: *Toll-like receptors cascades* (i.e. TLR1:TLR:2; TLR5, TLR6:TLR2, TL57/TLR8) and various cytokines signaling pathways (*i.e.* IL-1, IL-4, IL-10, IL-13). In addition, four pathways of the adaptive immune responses were upregulated, of which the *ER-phagosome pathway*, involved in antigen-processing cross presentation. Regarding signal transduction, Reactome GSEA particularly revealed gene networks upregulated in the G-alpha, NOTCH, MET, and Estrogen-dependent signaling cascades. In the cell cycle category, Reactome GSEA divulged up- and down-regulated pathways associated to the mechanism of cell cycle progression, such as *cell cycle checkpoints*, *mitosis*, and c*hromosome maintenance* (**Figure 4**). Regarding the metabolism, Reactome GSEA revealed additional categories containing upregulated pathways, such as the integration of *energy metabolism*, *metabolism of amino acids*, *metabolism of lipids*, *amyloid fiber formation* and *post-translational protein modifications*. Some of them have been very recently associated to *S. aureus* infection, such as a pathway associated to the metabolism of arachidonic acid that reportedly kills *S. aureus* through a lipid peroxidation mechanism (Beavers et al., 2019).

The Reactome GSEA also highlighted a gene cluster belonging to functional networks that were not identified with KEGG, in particular those associated with DNA repair or to gene expression, such as *epigenetic regulation*, *gene silencing by RNA*, *RNA polymerase I transcription*, *and RNA* polymerase *II transcription*, as well as *chromatin organization*. Among downregulated pathways the *neuronal system* pathway, with a cluster of 87 DEGs (the second pathway with the highest number of DEGs) was identified. In addition to their main role in neurotransmission, some genes are important in the regulation of bone metabolism (Bliziotes et al., 2001) and in the development of infection (Kim et al., 2018). Other downregulated pathways belonging to *Synthesis of DNA*, *collagen formation*, *degradation of extracellular matrix* (ECM), *integration of energy metabolism* and *signaling by receptor tyrosine kinase* (PTK2) categories were identified.

### Immune System and Signal Transduction Genes Are Among the Top Highly Induced DEGs

GSEA using both KEGG and Reactome databases pointed to the importance of changes in signal transduction and immune system-associated gene networks during the long-term *S. aureus* intracellular infection. Genes belonging to these functional categories represented 22% of DEGs associated to the KEGG hierarchy (504/2850) (**Table S2**). Moreover, they also accounted for 58% of the top highly regulated genes associated to the KEGG hierarchy (n = 64/194), with a clear bias toward upregulation (i.e., 57 DEGs with FC > 8 for only 7 DEGs FC < - 8). Among the upregulated genes there are genes that belong to infection-associated inflammatory Acute Phase proteins (APPs), such as Serum amyloid A1 (SSA1, FC = 1599) and A2 (SSA2, FC = 49), colony stimulating factor CSF2 (FC = 1316) and CSF3 (FC = 234), as well as Ceruloplasmin (CP; FC = 46.49) (**Table 2**).

**Table 2:** Immune system and signal transduction genes.

UniProt ID	Gene name	Gene description	FC	Log2FC	Adj.p-value
		**Upregulated genes**			
P0DJI8	*saa1*	serum amyloid A1	1599.72	10.64	2.93E-21
P04141	*csf*	colony stimulating factor 2 (granulocyte-macrophage)	1316.85	10.36	5.05E-39
P78556	*ccl20*	chemokine (C-C motif) ligand 20	396.66	8.63	7.36E-10
P09919	*csf3*	colony stimulating factor 3	234.28	7.87	6.34E-07
P80162	*cxcl6*	chemokine (C-X-C motif) ligand 6	174.86	7.45	1.67E-68
P10145	*cxcl8*	chemokine (C-X-C motif) ligand 8	134.94	7.08	2.42E-08
O95760	*il33*	interleukin 33	119.35	6.70	3.51E-11
P80098	*ccl7*	chemokine (C-C motif) ligand 7	99.50	6.63	8.09E-07
P25942	*cd40*	CD40 molecule, TNF receptor superfamily member 5	98.56	6.62	2.20E-21
P78423	*cx3cl1*	chemokine (C-X3-C motif) ligand 1	54.23	5.76	4.36E-05
P0DJI9	*saa2*	serum amyloid A2	48.93	5.61	1.58E-04
P02778	*cxcl10*	chemokine (C-X-C motif) ligand 10	48.42	5.60	7.40E-11
P00450	*cp*	ceruloplasmin (ferroxidase)	46.49	5.54	7.77E-08
O43557	*tnfsf14*	tumor necrosis factor superfamily member 14	45.01	5.49	2.24E-03
P05231	*il6*	interleukin 6	44.68	5.48	7.29E-10
P01584	*il1b*	interleukin 1 beta	40.45	5.34	8.06E-74
P09341	*cxcl1*	chemokine (C-X-C motif) ligand 1 (melanoma growth stimulating activity, alpha)	32.35	5.02	1.04E-03
P04003	*c4bpa*	complement component 4 binding protein, alpha	26.61	4.73	9.37E-08
P04233	*cd74*	CD74 molecule, major histocompatibility complex, class II invariant chain	25.50	4.67	1.57E-09
P04222	*hla-c*	major histocompatibility complex, class I, C	24.89	4.64	1.96E-04
P50591	*tnfsf10*	tumor necrosis factor superfamily member 10	22.27	4.48	1.22E-03
P30490	*hla-b*	major histocompatibility complex, class I, B	20.83	4.38	2.09E-05
P28907	*cd38*	CD38 molecule	18.88	4.24	5.94E-05
P24001	*il32*	interleukin 32	16.83	4.07	5.00E-39
P01583	*il1a*	interleukin 1 alpha	14.75	3.88	6.43E-13
O60603	*tlr2*	toll-like receptor 2	14.53	3.86	1.13E-25
P04179	*sod2*	superoxide dismutase 2, mitochondrial	14.24	3.83	8.07E-26
Q96RQ9	*il4i1*	interleukin 4 induced 1	12.60	3.66	3.81E-32
P08571	*cd14*	CD14 molecule	10.99	3.46	3.78E-37
Q13007	*il24*	interleukin 24	10.63	3.41	2.83E-02
O14862	*aim2*	absent in melanoma 2	10.13	3.34	1.22E-07
		**Downregulated genes**			
P29460	*il12b*	interleukin 12B	0.05	- 4.45	1.52E-03
Q99983	*omd*	periostin, osteoblast specific factor	0.05	- 4.31	6.97E-03
Q15063	*postn*	osteomodulin	0.11	- 3.16	1.47E-03
O60437	*ppl*	periplakin	0.15	- 2.77	9.06E-08

Genes encoding chemokines, chemiokines and inflammatory cytokines, which participate in the recruitment and activation of immune cells, were also highly upregulated, for instance: *CCL20*, *CXCL6*, *CXCL8*, *CCL7*, *CX3CL1*, *CXCL10*, and *CXCL1* (32<FC<396); *IL-33*, *IL-32*, *IL-6*, *IL-1β*, *IL-1α*, *IL-24* (10<FC<119), and TNF-family members (*TNFSF10* and *TNFSF14* with a FC of 22 and 45, respectively). Moreover, genes encoding extracellular receptors, such as a Toll-like receptor 2 (*TLR2*, FC = 15), and the mitochondrial enzyme superoxide dismutase-2 (*SOD2*, FC = 14) that are involved in the killing of internalized pathogenic bacteria (Abuaita et al., 2018) were highly upregulated. The expression of Absent in Melanoma 2 (*AIM2*) that induces an activation of immune signaling platforms known as inflammasomes (Broz and Dixit, 2016) was also increased (FC = 10). Other upregulated genes of immune system and signal transduction categories are listed in **Table 2** and **Table S2**. Among the most downregulated DEGs were the interleukin 12B gene (FC = 0.046), which plays a protective role during bacterial infection (Reeme et al., 2013), the osteoblast-specific factor-2, periostin (FC= 0.112), as mentioned above, osteomodulin (FC=0.05) that functions as a positive coordinator in osteogenesis (Lin et al., 2020) and periplakin (FC=0.147), which connects cytoskeletal structures to the cell adhesion complex (Hu et al., 2018).

### *S. aureus* Infection Triggers Transcriptional Reprogramming of Genes Involved in Metabolism

The second group of the top highly regulated genes and enriched pathways highlighted with both KEGG and Reactome databases belongs to the metabolism category (291 genes including 22 top-highly upregulated genes, **Table S2**) and signal transduction and immune system categories genes that also play an important role in metabolism. Among them the APP *SAA2* (FC = 49, as mentioned above), is involved in high density lipoproteins metabolism and cholesterol homeostasis, (Ely et al., 2001; Krishnan et al., 2015), the phospholipases A2 (FC = 75) is implicated in lipid metabolism, and the hydroxysteroid (11-beta) dehydrogenase 1 (FC = 53) is involved in hormone metabolism (**Table 3**).

**Table 3:** Metabolism genes.

UniProt ID	Gene name	Gene description	FC	Log2FC	Adj.p-value
		**Upregulated genes**			
P14555	*pla2g2a*	phospholipase A2 group IIA	75.85	6.25	3.52E-03
P28845	*hsd11b1*	hydroxysteroid (11-beta) dehydrogenase 1	53.22	5.73	3.69E-04
134339	*saa2*	serum amyloid A 2	48.93	5.61	1.58E-04
P00167	*cyb5a*	cytochrome b5 type A	24.61	4.62	7.45E-05
P04179	*sod2*	superoxide dismutase 2. mitochondrial	14.24	3.83	3.17E-23
Q8TDS4	*hcar2*	hydroxycarboxylic acid receptor 2	13.22	3.72	5.34E-07
C9JRZ8	*akr1b15*	aldo-keto reductase family 1	10.51	3.39	1.21E-02
A1L3X0	*elovl7*	ELOVL fatty acid elongase 7	7.70	2.94	1.68E-06
O95992	*ch25h*	cholesterol 25-hydroxylase	6.26	2.65	3.36E-03
P43490	*nampt*	nicotinamide phosphoribosyltransferase	5.51	2.46	6.99E-56
Q9H2J7	*slc6a15*	solute carrier family 6 member 15	4.56	2.19	3.76E-09
Q99541	*plin2*	perilipin2	4.46	2.16	2.42E-23
Q9NXB9	*elovl2*	ELOVL fatty acid elongase 2	3.56	1.83	2.06E-19
Q9Y5L2	*hilpda*	hypoxia inducible lipid droplet-associated	3.16	1.66	5.29E-13
***glycolysis genes***					
		**Upregulated genes**			
Q9BYZ2	*ldhal6b*	lactate dehydrogenase A-like 6B	6.52	2.71	2.74E-02
Q6PCE3	*pgm2l1*	phosphoglucomutase 2-like 1	4.37	2.13	9.55E-19
P06733	*eno1*	Enolase, phosphopyruvate hydratase	1.79	0.84	1.22E-02
P00338	*ldha*	lactate dehydrogenase A	1.67	0.74	5.45E-02
P04075	*aldoa*	aldolase, fructose-bisphosphate A	1.66	0.73	8.12E-03
		**Downregulated genes**			
P08237	*pfkm*	phosphofructokinase	0.69	- 0.54	2.29E-02
O43175	*phgdh*	phosphoglycerate dehydrogenase	0.61	- 0.72	4.48E-02

The expression of *SOD2* that protects the host against reactive oxygen and reactive nitrogen species (Eisenreich et al., 2019), but is also involved in metabolic reprogramming in gastric cancer (Liu et al., 2019) was also increased (FC = 14). Besides, we observed the high level of expression of a hydroxycarboxylic acid receptor 2 (*HCA2*, FC= 13), which regulates lipolysis and at the same time reduces pro-inflammatory cytokines level in sepsis (Takakura and Zandi-Nejad, 2019). Moreover, we found other upregulated genes with lower FC, which are involved in metabolic processes such as cholesterol 25-hydroxylase (*CH25H*, FC = 6), an interferon-stimulated gene that converts cholesterol to the oxysterol 25-hydroxycholesterol (Abrams et al., 2020), fatty acid elongases 2 and 7 (*ELOVL2* and *ELOVL7* with FC= 3.5 and 7.7, respectively) involved in synthesis of long-chain saturated fatty acids (Jump, 2009), and perilipin 2 (FC = 4.5), a protein belonging to the family of cytoplasmic lipid droplet binding protein that can be used by osteoblasts as a fuel source (Rendina-Ruedy et al., 2017).

RNA-seq analysis also pointed to the deregulation of genes coding for enzymes of glycolysis and gluconeogenesis such as lactate dehydrogenase A-like 6B (FC = 6.523), which catalyzes the conversion of pyruvate into lactic acid and phosphoglucomutase (FC = 4.374) that facilitates the interconversion of glucose 1-phosphate and glucose 6-phosphate (**Table 3**). Additionally, to the above listed genes, there are other metabolism-associated genes, which likely provide a source of nutrients, energy, and metabolites that promote bacterial intracellular survival and proliferation.

### *S. aureus* Infection Triggers Transcriptional Reprogramming of Genes Involved in Neurotransmission

Reactome GSEA underlined the downregulation of the neuronal system pathway. DEGs included gamma-aminobutyric acid (*GABA*) type A receptor subunit alpha2 (*GABRA2*, FC = 9.2) and alpha3 (*GABRA3*, FC = 0.3); 5-hydroxytryptamine (serotonin) receptor 4 (*HTR4*, FC = 7.3) and 2A (*HTR2A*, FC = 5.9), as well as glutamate ionotropic receptor N-methyl D-aspartate type subunit 2A and 3A (*GRIN2A* and *GRIN3A* with FC = 7 and 0.3, respectively). Gene coding for 4-aminobutyrate aminotransferase (*ABAT*, FC = 0.3) that is responsible for catabolism of GABA, and gene coding for calcitonin-related polypeptide beta (*CALCB*, FC = 0.14), a highly potent vasodilator were down-regulated (Russell et al., 2014) (**Table 4**).

**Table 4:** Neurotransmitter genes.

UniProt ID	Gene name	Gene description	FC	Log2FC	Adj.p-value
		**Upregulated genes**			
P47869	*gabra2*	gamma-aminobutyric acid (GABA) A receptor, alpha 2	9.20	3.20	7.47E-11
Q13639	*htr4*	5-hydroxytryptamine (serotonin) receptor 4, G protein-coupled	7.30	2.87	1.74E-02
Q12879	*grin2a*	glutamate receptor, ionotropic, N-methyl D-aspartate 2A	7.10	2.83	4.08E-04
P28223	*htr2a*	5-hydroxytryptamine (serotonin) receptor 2A. G protein-coupled	5.88	2.56	4.42E-03
		**Downregulated genes**			
P34903	*gabra3*	gamma-aminobutyric acid (GABA) A receptor, alpha 3	0.33	- 1.61	1.68E-04
P80404	*abat*	4-aminobutyrate aminotransferase	0.29	- 1.80	4.51E-03
Q8TCU5	*grin3a*	glutamate receptor, ionotropic, N-methyl-D-aspartate 3A	0.28	- 1.86	2.22E-03
P10092	*calcb*	calcitonin-related polypeptide beta	0.14	- 2.88	2.87E-04

Other deregulated genes encoding neurotransmitter-associated protein were identified in infected MG-63 cells (**Table S1**).

### *S. aureus* Infection Triggers Transcriptional Reprogramming of Genes Involved in Epigenetic Regulation

Reactome GSEA revealed deregulation of pathways related to epigenetic modifications/regulations. The selective activation or repression of specific genes not only depends on transcription factors, but also on their interaction with epigenetic modulators (or “epifactors”), which regulate DNA accessibility by controlling the structure of chromatin. Epigenetic modifications of chromatin include DNA methylation and hydroxymethylation, as well as multiple histones post-translational modifications, such as acetylation, methylation, phosphorylation, ubiquitylation, serotonylation and dopaminylation (Chan and Maze, 2020). We sought to determine which DEGs encoded epifactors and whether they were up- or down-regulated. To do this, we intersected either the 1514 upregulated DEGs or the 1336 downregulated DEGs associated with *S. aureus* intracellular infection with the Epifactor database (Medvedeva et al., 2015). This database includes 720 epifactors classified according to their function: (i) enzymes that “write” epigenetic marks, such as DNA methyltransferases (DNMTs) and histone acetyltransferases (HATs) and methyltransferases (HMTs), (ii) enzymes that “erase” epigenetic marks, such as histone deacetylases (HDACs) and demethylases (HDMs); (iii) proteins that “read” these marks, (iv) chromatin-remodeling enzymes that displace nucleosomes, (v) scaffold proteins that assemble macromolecular chromatin-regulatory complexes, and (v) diverse cofactors. This analysis identified an important number of DEGs encoding epifactors (117 DEGs), of which 92 were downregulated (**Table S6**) and 25 upregulated (**Table S7**).

The strikingly important number of epifactor genes which were downregulated by infection (7% of all of the downregulated DEGs) prompted us to examine their functions in detail, using the Gene Ontology of Biological Processes (GO-BP) enrichment analysis of the DAVID software. This analysis showed that 30 of these genes encoded epifactors with a negative effect on transcription (*i.e.*, repressors): *BAHD1, BRCA1, CBX1, CBX2, CBX6, CBX5, CHD4, CTCF, DNMT1, DNMT3A, EHMT1, GATAD2A, HDAC4, HDAC6, HDAC10, KDM5C, MBD1, MBD3, NSD1, PARP1, PRMT6, RCOR1, SCMH1, SIN3B, SMARCA4, TRIM24, UHRF1, WHSC1, ZGPAT, ZMYND11* (**Table S7**). Interestingly, several of these components belong to chromatin-repressive complexes, in particular, the BAHD1 (4 genes), NurD (4 genes), Polycomb PRC1 (5 genes), mSin3A (1 gene) and CoREST (1 gene) complexes (**Table 5** and **Table S5**). Among other downregulated DEGs falling into the epifactor category, we noticed several gene coding for histone deacetylases (*HDAC4*, *HDAC6*, *HDAC10*), and components of the DNA methylation and demethylation pathways. The later included writers (i.e. the “*de novo*” methyltransferase DNMT3a, the “maintenance” methyltransferase *DNMT1*, and the Tet1 methylcytosine dioxygenase), readers (the methyl-CpG binding domain protein 1 and protein 3, *MBD1*, *MBD3*), and cofactor *UHRF1* (which promotes DNMT1 action). Of note, MBD1 and MBD3 belong to different chromatin-repressive complex that link histone modification to DNA methylation, such as BAHD1 and NurD. Other examples of downregulated epigenetic gene writers were the euchromatic histone-lysine N-methyltransferase 1 (*EHMT1*) and lysine (K)-specific methyltransferase 5A (*KMT5A*). Expression of readers that recognize methyl-lysine residues was also altered. Among them the expression of members of the splindin family (*SPIN1* and *SPIN4*) was down regulated.

**Table 5:** Epigenetics genes.

UniProt ID	Gene name	Gene description	FC	Log2FC	Adj.p-value value
		**Upregulated genes**			
Q9C005	*dpy30*	dpy-30,histone methyltransferase complex regulatory subunit	1.59	0.67	9.18E-03
Q7Z2T5	*trmt1l*	tRNA methyltransferase 1 like	1.52	0.60	1.80E-03
Q9BVS5	*trmt61b*	tRNA methyltransferase 61B	1.52	0.60	1.61E-02
Q96T68	*setdb2*	SET domain, bifurcated 2	1.45	0.54	3.76E-02
Q9Y657	*spin1*	splindlin 1	0.70	-0.53	2.22E-02
		**Downregulated genes**			
		**NuRD complex**			
Q13330	*mta1*	metastasis associated 1	0.66	-0.61	4.54E-02
Q86YP4	*gatad2a*	GATA zinc finger domain containing 2A	0.53	-0.93	1.51E-02
Q14839	*chd4*	chromodomain helicase DNA binding protein 4	0.70	-0.52	1.25 E-02
O95983	*mbd3*	methyl-CpG binding domain protein 3	0.68	-0.55	3.88E-02
		**BAHD1 complex**			
Q8TBE0	*bahd1*	bromo adjacent homology domain containing 1	0.68	-0.55	7.35E-03
P83916	*cbx1* *(hp1-beta)*	chromobox homolog 1 (Heterochromatin protein 1 homolog beta)	0.71	-0.50	2.32E-02
P45973	*cbx5* *(hp1-alpha)*	chromobox homolog 5 (Heterochromatin protein 1 homolog alpha)	0.51	-0.96	2.88E-02
Q9UIS9	*mbd1*	methyl-CpG binding domain protein 1	0.65	-0.62	1.14E-02
		**Polycomb repressive complex 1 (PRC1)**			
Q92560	*bap1*	BRCA1 associated protein-1	0.62	-0.69	5.95E-04
Q14781	*cbx2*	chromobox homolog 2	0.49	-1.01	9.03E-06
O95503	*cbx6*	chromobox homolog 6	0.39	-1.37	8.35E-08
Q96GD3	*scmh1*	sex comb on midleg homolog 1 (Drosophila)	0.71	-0.49	4.63E-02
Q9UQR0	*scml2*	sex comb on midleg-like 2 (Drosophila)	0.58	-0.78	7.94E-03
		**DNA methyltransferases**			
P26358	*dnmt1*	DNA (cytosine-5-)-methyltransferase 1	0.60	-0.87	1.63E-02
Q9Y6K1	*dnmt3a*	DNA (cytosine-5-)-methyltransferase 3 alpha	0.46	- 1.10	4.01E-02
		**Methylcytosine dioxygenase**			
Q8NFU7	*tet1*	tet methylcytosine dioxygenase 1	0.58	-0.78	1.02E-03
		**Histone deacetylases**			
P56524	*hdac4*	histone deacetylase 4	0.57	-0.80	1.02E-02
Q9UBN7	*hdac6*	histone deacetylase 6	0.63	-0.67	1.09E-04
Q969S8	*hdac10*	histone deacetylase 10	0.29	- 1.81	2.59E-02
		**Histone methyltransferases**			
Q9H9B1	*ehmt1*	euchromatic histone-lysine N-methyltransferase 1	0.56	-0.84	5.37E-03
Q9NQR1	*kmt5a*	lysine (K)-specific methyltransferase 5A	0.57	-0.80	3.65E-06
		**Others**			
Q9Y657	*spin1*	spindlin family member 1	0.69	-0.53	2.22E-02
Q56A73	*spin4*	spindlin family member 4	0.47	- 1.10	1.76E-05
Q96T88	*uhrf1*	ubiquitin-like with PHD and ring finger domains 1	0.45	- 1.15	1.38E-03

## Discussion

**Figure 5:**
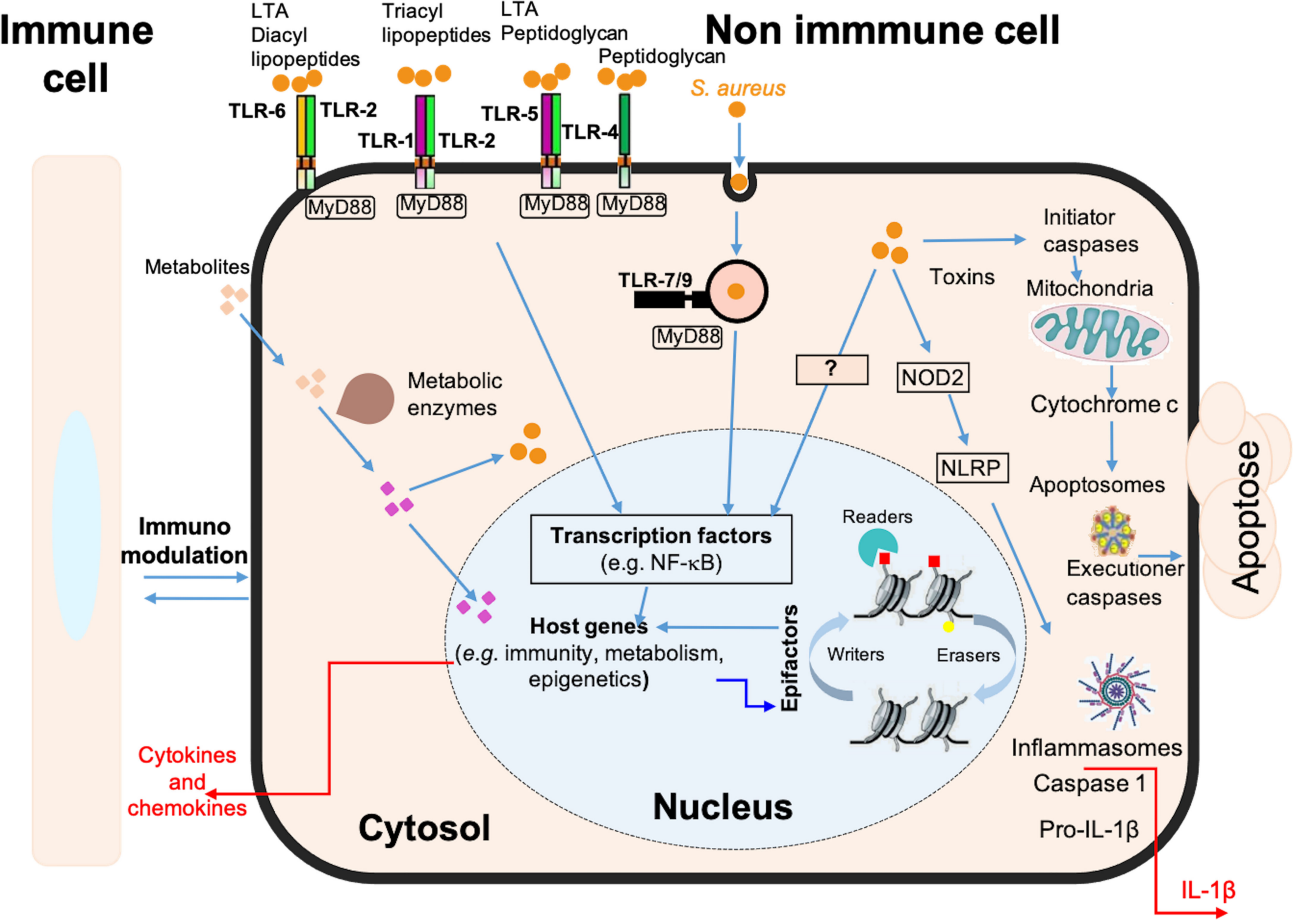
Schematic model of the immune, metabolic and epigenetic dysregulated signatures induced by long-term *S. aureus* infection in osteoblasts. Pattern recognition receptors, such as Toll-like receptors (e.g. TLR-1, -2, -4, -5, -6, -7, -9) and NOD-like receptors (e.g. NOD2), are involved in the detection of bacteria (*S. aureus*, represented by orange dots). These receptors trigger intracellular signaling cascades, resulting in the activation of transcription factors, such as NF-κB, and the up-regulation of genes encoding immune and inflammatory proteins, such as cytokines, chemokines, and components of the inflammasome. These responses are also dependent on the chromatin-dependent regulation of gene expression controlled by epigenetic factors (i.e., epifactors), such as writers, erasers, and readers of chromatin modifications (red and yellow squares), as well as by metabolic pathways. Many genes encoding epifactors with repressive activity are inhibited by infection by an unknown signaling cascade (represented by a question mark). Putative metabolites that are taken up by cells (pink squares) or that are processed internally by metabolic enzyme activity (purple squares) have effects on cytosolic responses, or can enter the nucleus and act on chromatin modifications, epifactors or epigenetic genes expressions. Host metabolism influences *S. aureus* survival. Bacteria can also produce effectors that impact these processes. These interactions determine the magnitude of the host response to infection, including the immune response, inflammatory reactions, cell death, DNA damages, as well as immunomodulation of neighboring cells.

Osteomyelitis has so far been little studied by transcriptomic approaches and, to our knowledge, none has yet focused on the impact of intracellular *S. aureus* infection in osteoblasts (Hofstee et al., 2020). Here, we present a new model to study a late intracellular life stage of *S. aureus* in osteoblast-like cells. In the absence of signals from uninfected cells, the transcriptomic analysis presented here identifies genes, signaling pathways and cellular processes specifically impacted by intracellular bacteria (**Figure 5**). The results suggest an important role of osteoblasts in the inflammatory phenomena observed during *S. aureus-associated* osteomyelitis, due to the activation of a large network of innate immunity genes. This activation is concomitant with the inhibition of numerous genes coding for epifactors involved in chromatin-dependent transcriptional inhibition. In addition, infection alters the expression of a set of metabolic genes that may affect bacterial survival, as well as genes encoding neurotransmitters and cell adhesion proteins.

### A Potent Immune Response During Long-Term *S. aureus* Infection in Osteoblasts

The signature of the infection is first characterized by the activation of a very large network of innate immune response genes in osteoblast-like cells infected for 6 days. Although expected, this defense response is remarkable for its amplitude. Among the induced genes, those encoding secreted factors (about 200) may serve as markers in the early diagnosis of infection and in the differentiation of infectious and non-infectious causes of osteomyelitis. Among the highly induced genes are genes encoding acute phase proteins (APPs), such as *SSA1* and *SSA2*, which are involved in innate immunity and lipid homeostasis during inflammation (Zheng et al., 2020), CP, encoding ceruloplasmin, the major blood copper transport protein, which also plays a role in iron metabolism. While the hepatic synthesis of APPs is well established, their extrahepatic expression remains a matter of debate. Strong expression of several APPs by osteoblast-like cells in response to intracellular *S. aureus* infection is therefore noteworthy. Osteoblasts could be APP-secreting cells specifically in bone tissue, as also predicted by the detection of APPs in a porcine model of osteomyelitis and in material from human patients with chronic osteomyelitis (Lüthje et al., 2020). We also report increased expression of many genes encoding cytokines and chemokines, such as CCL2, CCL5, CCL7, CCL8, CCL10, CCL11, CCL13, CCL20, CCL26, and CXCL1, CXCL2, CXCL3, CXCL5, CXCL6 and *CX3CL1*, which are known to be immune cell chemoattractants (Hieshima et al., 1997; Sokol and Luster, 2015). *CXCL6* and *CXCL8* have recently been proposed for the detection of inflammation during bone disease (Grad et al., 2016; Afzelius et al., 2020).

The immune system category also includes genes revealing an activation of inflammasomes (Schroder and Tschopp, 2010) whose aberrant activation is downregulated by mitophagy (Kim et al., 2016). The involvement of inflammasome-mediated IL-1β in *S. aureus* clearance in a mouse skin infection model (Miller et al., 2007) and in a short-term infection of osteoblastic cells was previously demonstrated by our team (Lima Leite et al., 2020). The increased expression of IL-1β and inflammasome-associated AIM2 as well as an implication of the *Mitophagy pathway* reported here supports the involvement of inflammasomes in the long-term infection leading to the development of osteomyelitis. We also highlight numerous genes of other upregulated cytokines, such as IL-1α, *IL-6*, *IL-15*, *IL1-18*, *IL-24*, *IL-32*, *IL-33*, *IL-34* and *IFN-β*, and TNFs. It will be interesting to test the involvement of these aforementioned chemokines and cytokines in *S. aureus* osteomyelitis and their use as infection markers. Additionally, we report a deregulation of the *Complement and coagulation cascades* pathway. Since a complement system is involved in the restriction of the growth of internalized bacteria by autophagy (Sorbara et al., 2018) and a detection of deregulated autophagy-related genes (*ATG2*, *ATG5*, *ATG7*, *ATG9*) in our model, further investigations are required for the understanding of the complement system and autophagy relationship in the context of osteomyelitis.

### A Network of Epifactor Genes Is Deregulated by Long-Term Infection With *S. aureus*

The transcriptional activation of innate immunity and inflammation genes observed in this response of osteoblasts to *S. aureus* is associated with the activation of numerous genes coding for signaling transduction proteins and transcription factors known to activate these genes (e.g., TNF-, NF-κB, TLR-, NOD-like receptor-, Jak-STAT-, cytosolic DNA sensing- signaling pathways). The amplitude of the expression could, however, be also related to the deregulation of genes involved in chromatin remodeling. Beside immunity, infection-mediated deregulation of genes belonging to the *estrogen signaling* and *osteoblast differentiation pathways* could also be due to chromatin-based regulation (Kovats, 2015; Montecino et al., 2021). The expression of cellular genes is indeed dependent on the state of chromatin compaction, governed by histone modification and DNA methylation profiles, also referred to as “epigenetic regulation”. A growing number of studies have shown that infections with different pathogens can profoundly alter host epigenetic information, either by promoting alterations in epigenetic marks or by deregulating epifactors (i.e., writers, readers and erasers of epigenetic marks, scaffolding proteins, and coregulators) (Bierne et al., 2012; Bierne, 2017; Fischer, 2020). The role of epigenetic regulations in the development of *S. aureus-associated* osteomyelitis has not yet been studied. Here, we found that the expression of a significant number of epifactor-encoding genes is altered upon long-term intracellular infection with *S. aureus*, primarily through down-regulation, and importantly, that a majority of them encode epifactors acting as repressors. To our knowledge, this is the first report describing such a massive effect of intracellular bacterial infection on the epifactor gene network. This inhibition of chromatin repression mechanisms may play an important role in the concomitant and potent activation of genes associated with inflammation and immunity by overcoming transcriptional blocks at target genes. These epigenetic mechanisms include components of macromolecular chromatin-repressive complexes, such as NurD (Xue et al., 1998, Denslow et al., 2007), Polycomb-repressive complex PRC1 (Parreno et al., 2022), and the recently described BAHD1 complex (Bierne H. et al., 2011; Lakisic et al., 2016). Interestingly, BAHD1 and NurD are known to be controlled by the pathogens *Listeria monocytogenes* and *Mycobacterium tuberculosis*, (Lebreton et al., 2012; Olias et al., 2016), resulting in deregulation of interferon responses (Lebreton et al., 2011). However, this control does not occur through mechanisms involving changes in the expression of genes encoding subunits of these macromolecular complexes. The infection-mediated deregulation of these epifactor genes that we have observed here is, in this respect, novel.

Attention should also be drawn to the downregulation of the histone methyltransferase genes *EHMT1* and *KMT5A*, as these factors are also involved in epigenetic repression of transcription. For example, *EHMT1* functions as a negative regulator in NF-κB and type I interferon-mediated gene induction pathways (Ea et al., 2012). Down-regulation of *EHMT1* can enhance the expression of a subset of NF-ĸB-regulated genes and increase interferon production, which is essential for immunity against *S. aureus* infection (Parker and Prince, 2012). Recently, it has been shown that inhibition of *KMT5A*, which is involved in multiple biological processes, suppresses key molecules involved in lipid metabolism (Liao et al., 2018).

Several genes encoding proteins involved in DNA and histone methylation, as well as histone deacetylation, are also downregulated in infected MG-63 cells. In particular, the expression of the major DNA cytosine methyltransferases (*DNTM3A* and *DNMT1*) was significantly downregulated. This suggests that long-term infection disrupts *de novo* methylation (*via* DNMT3A gene deregulation) and maintenance of methylation during cell proliferation (*via DNMT1* gene deregulation) (Wienholz et al., 2010; Ren et al., 2020). In addition, down-regulation of *UHRF1*, *MBD1* and *MBD3* has also been observed. UHRF1, plays a major role in maintaining DNA methylation, as it binds to hemi-methylated CpGs during replication and enables the action of *DNMT1*. MBD1 and MBD3, which bind to methyl-CpGs, promote chromatin condensation and gene silencing (Valinluck et al., 2004; Liu et al., 2013b). All of these changes could result in hypomethylation of certain loci and lasting imprints of infection. Hypomethylation may also induce genomic instability (Pappalardo and Barra, 2021). We also observed down-regulation of several HDACs (*HDAC4*, 6, and 10) suggesting decreased histone deacetylation, which is essential for histone-DNA interaction. Altered acetylation of repeat regions also promotes genome instability (Gershon and Kupiec, 2021) in addition to the effect of DNA hypomethylation. Downregulation of the splindin1 and splindin4 genes encoding readers recognizing histone H4K20me3 methylation, which is a hallmark of silenced heterochromatic regions and associated with DNA replication and repair (Wang et al., 2018), may also play a role in genome instability. Overall, it would be important to examine in the future whether *S. aureus* alters DNA methylation, acetylation, and histone methylation patterns in osteoblasts, as these epigenetic modifications could profoundly reprogram the host cell in the long term, beyond infection, leading to secondary effects in chronic infections (Bierne et al., 2012; Bierne, 2017).

### Impact of Infection on Host DNA Integrity

We have previously shown that *S. aureus* induces a delayed G2/M phase transition, associated with increased intracellular bacterial replication. Furthermore, this phenomenon causes DNA damage in host cells (Alekseeva et al., 2013; Deplanche et al., 2015; Deplanche et al., 2019). Reactome GSEA highlighted 7 enriched pathways that belong to cell cycle progression, including those associated with kinetochores and telomeres. In addition, the expression of many genes involved in DNA repair is inhibited by infection. *S. aureus-*induced DNA damage could contribute to senescence of infected cells, particularly induced by telomere stress, which is consistent with the current view of the role of senescence during infections (Humphreys et al., 2020). But if infected cells escape senescence, incomplete DNA damage repair could have a mutagenic effect. Thus, it is important to consider the potential impact of intracellular *S. aureus* infection on the genome and epigenome integrity of osteoblasts.

### Long-Term Infection With *S. aureus* Induces Changes in the Expression of Metabolic Genes

The shift from oxidative phosphorylation to aerobic glycolysis in host cells is crucial for a response during infection (O’Neill and Pearce, 2016). In particular, glycolysis is required for *S. aureus* survival in an osteomyelitis model and activation of glycolysis by SCVs triggers necroptosis of infected cells, leading to the release of viable staphylococci (Wickersham et al., 2017; Palma Medina et al., 2019; Potter et al., 2020; Wong Fok Lung et al., 2020). Glycolysis stabilizes the transcription factor HIF-1α and increases IL-1β expression, linking metabolic and immune responses during infection (Tannahill et al., 2013). Here, KEGG analysis revealed altered host *glycolysis/glycogenesis* pathway, induction of *necroptosis*, involvement of the *HIF-1α pathway*, and increased IL-1β production. This suggests that in addition to promoting bacterial growth, the alteration of glycolysis switches the maintenance activities of osteoblasts to defense processes. The final outcome of the infection may therefore depend on the balance between these modalities.

Pathogens utilize host lipids/lipoproteins, including fatty acids, to enable their proliferation (Feingold and Grunfeld, 2012; Morvan et al., 2016; Lopez et al., 2017). Unsaturated fatty acids from the host are incorporated into *S. aureus* membranes, leading to a decrease in bacterial membrane fluidity and activation of the type VII secretion system, dedicated to the export of virulence factors, which promotes bacterial persistence (Lopez et al., 2017). The functional diversity of fatty acids depends on their chain length and degree of unsaturation that is determined in the elongation process. The enzymes ELOVL1, 3, 6, 7 elongate saturated and monounsaturated fatty acids, whereas ELOVL2, 4, 5 elongate polyunsaturated fatty acids (Jump, 2009; Naganuma et al., 2011). Upregulation of *ELOVL2* and *ELOVL7* gene expression suggests the involvement of fatty acid elongation in the response of *S. aureus-*infected osteoblasts. Modulation of the balance between saturated and unsaturated fatty acids in the host could be involved in the outcome of the infection.

Host lipids are sequestered in lipid droplets, cytosolic lipid storage organelles, comprising a monolayer of phospholipids surrounding a hydrophobic core of neutral lipids: cholesterol esters and triacylglycerols. Our results identified the infection-mediated upregulation of genes encoding lipid droplet-associated molecules, including anti-lipolytic *HCAR2* (Offermanns, 2017), perilipin -2 and -3, which regulate lipid droplet formation and degradation (Libbing et al., 2019) and *HILPDA* (**Table 3**).

It was shown that *M. tuberculosis* activates HCAR2 in macrophages with subsequent accumulation of lipid droplets, which then provide the bacteria with fatty acids as nutrients (Singh et al., 2012). In addition, bacilli engulfed in lipid droplets decrease their replication and acquire phenotypic resistance to certain drugs (Daniel et al., 2011). To our knowledge, the role of HCAR2 during *S. aureus* infection has not been studied to date. Higher concentrations of intracellular triacylglycerol and larger lipid droplets were observed in *S. aureus-infected* adipocytes, which was partially attributed to a reduced rate of lipolysis (Hanses et al., 2011). These results, along with ours, suggest that HCAR2 may mediate the inhibition of lipolysis in *S. aureus-infected* cells and that lipid droplets may serve as a source of nutrients. Collectively, our results suggest that alterations in metabolism induced by *S. aureus* internalization, at least in part, may be favorable for bacterial persistence.

### Neurotransmitter Genes Are Perturbed by *S. aureus* Infection

The observation of deregulation of several genes of the *neuronal system pathway* in a bone-associated cell type was intriguing. It may be noted that glutamate and GABA signaling are known to act in the antibacterial response through the enhancement of autophagy (Hassel et al., 2014; Kim et al., 2018). The role of glutamate and GABA receptors therefore merits investigation in the context of *S. aureus* osteomyelitis. Release of the neurotransmitter serotonin by mast cells was demonstrated *in vivo* after an administration of staphylococcal enterotoxin (Hu et al., 2007). Moreover, the role of neurotransmitter was studied in other models of infection. Indeed, it was demonstrated that intestinal serotonin decreases virulence gene expression of enterohemorrhagic *E. coli* and *Citrobacter rodentium*, in a murine model (Kumar et al., 2020). Besides, it was established that the production or release of neurotransmitters upon bacterial infection controls immune response intensity in *C. elegans* (Masuzzo et al., 2020). Here our results suggests that serotonin might also be involved *S. aureus-*associated bone infections, based on the observation of upregulation of the serotonin receptor gene and transglutaminase 2 (TGM2). This, too, deserves further investigation, particularly in the light that a new class of post-translational modification, serotonylation, relies on the action of TGM2 (Bader, 2019; Farrelly et al., 2019; Chan and Maze, 2020). We are convinced that the deregulation of several genes of the neuronal system pathway during a long-term of *S. aureus* infection is in line with the recent findings described for other pathogens.

### Cell Adhesion and ECM-Associated Gene Networks Are Perturbed by *S. aureus* Infection

Secreted by cells into the extracellular space, ECM plays an pivotal role in *S. aureus* adhering to and invading non-phagocytic cells (Liang and Ji, 2006) and is involved in the development of the dormancy of intracellular *M. tuberculosis* (Arbués et al., 2021). Focal adhesion kinase PTK2 regulates adhesion of *S. aureus* to ECM, reorganization of the actin cytoskeleton, cell cycle progression, cell proliferation and apoptosis that may disturb the defensive barrier function of host cells (Hermann et al., 2015). Downregulation of *degradation of extracellular matrix* and *PTK2 signaling* pathways suggests further examination of ECM and PTK2 impact on intracellular life of *S. aureus*.

## Conclusion

**Figure 6:**
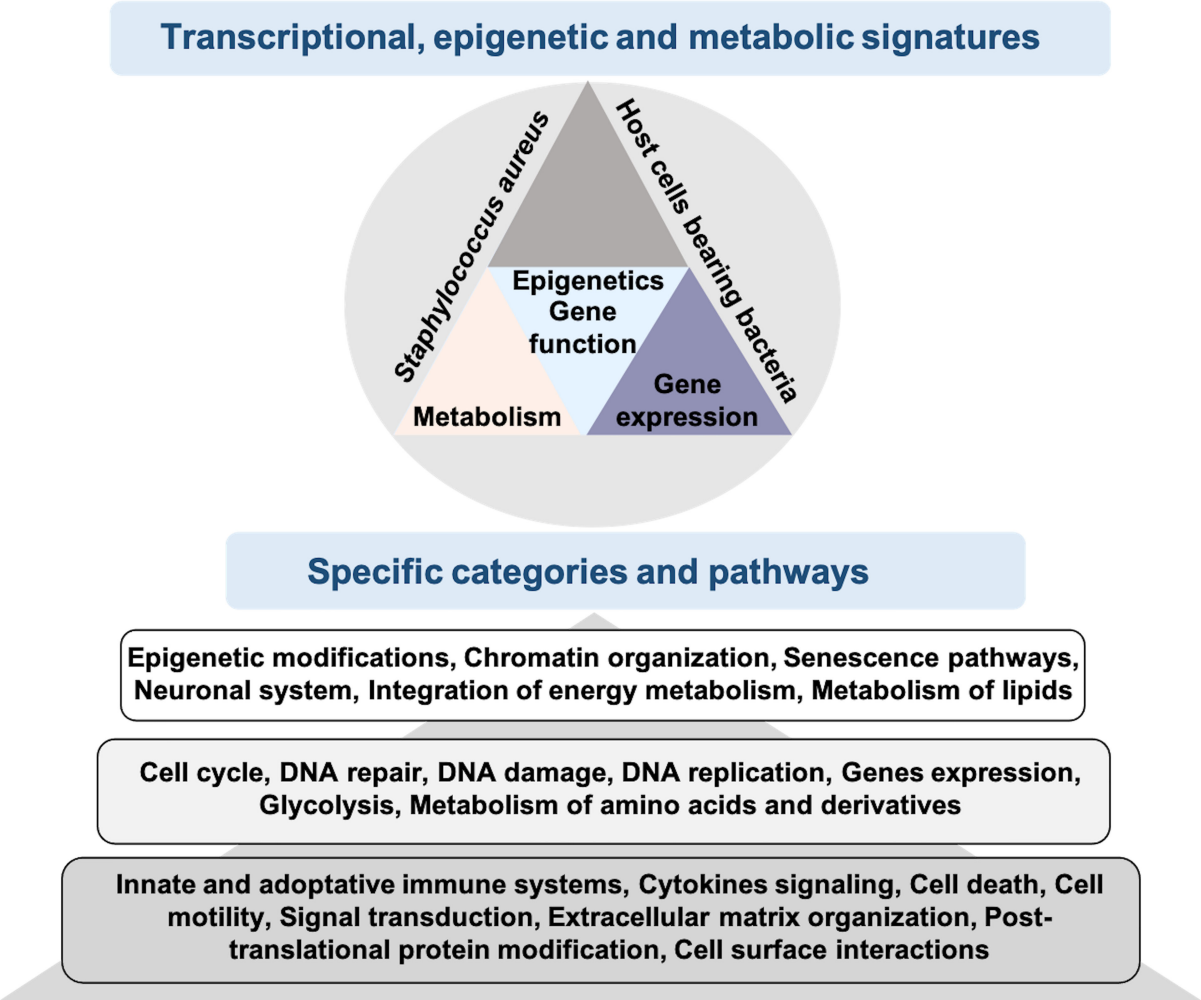
Results of RNA-seq of osteoblast-like cells bearing *S. aureus* promotes knowledge integration. RNA-seq analysis of infected cells bearing internalized *S. aureus* allows transcriptional, epigenetic and metabolic signatures to be obtained. Using KEGG and Reactome enriched pathways analysis of *S. aureus* bearing cells, we identified specific categories and pathways that are either well studied (dark gray), medium studied (gray) or mostly non-studied (light gray).

We provide here an atlas of genes and pathways deregulated by the intracellular presence of the pathogen *S. aureus* in an osteoblast model. This knowledge not only improve our conceptual understanding of biological processes involved in the long-term *S. aureus* infections, but also indicates the direction for future research and highlights potential candidates for the development of new diagnostic, prophylactic and therapeutic approaches. The deregulation of epigenetic and DNA repair pathways opens the hypothesis that intracellular *S. aureus* infection has a long-term impact on the genome and epigenome of host cells, which may exert patho-physiological dysfunctions additionally to the defense response during the infection process (**Figure 6**).

## Data Availability Statement

The datasets presented in this study can be found in online repositories. The name of the repository and accession number(s) can be found at: https://www.ebi.ac.uk/ena/browser/view/PRJEB47070.

## Author Contributions

Investigation and methodology: HB, FL, AN, MD, AD, CG, WM, P-HC, and NB. Formal analysis: AN, EG, HB, and NB. Visualization and data curation: AN, EG, and NB. Funding acquisition: PG, VA, HB, and NB. Writing - original draft preparation: AN, HB, and NB. Writing - review and editing: FL, VA, EG, YLL, HJ, HB, and NB. Supervision: NB. Conceptualization: HB and NB. Administration: NB. All authors contributed to the article and approved the submitted version.

## Funding

RNA sequencing experiments were performed in collaboration with the GeT core facility, Toulouse, France (http://get.genotoul.fr), and was supported by France Génomique National infrastructure, funded as part of “Investissement d’avenir” program managed by Agence Nationale pour la Recherche (contract ANR-10-INBS-09). WM was supported by Institutional Programme of Scholarships for Sandwich Doctorates Abroad CAPES-PDSE (number: 2890-13-5). NB, HJ, YL, MD, and PG were supported by a Metaprogram INRAE, GISA, LONGhealth–MPP10573. HB was supported by ANR (contracts ANR-20-CE35-0001 and ANR-20-PAMR-0011).

## Conflict of Interest

The authors declare that the research was conducted in the absence of any commercial or financial relationships that could be construed as a potential conflict of interest.

## Publisher’s Note

All claims expressed in this article are solely those of the authors and do not necessarily represent those of their affiliated organizations, or those of the publisher, the editors and the reviewers. Any product that may be evaluated in this article, or claim that may be made by its manufacturer, is not guaranteed or endorsed by the publisher.
