# Supplementary material for: Transcriptome Architecture of Osteoblastic Cells Infected With Staphylococcus aureus Reveals Strong Inflammatory Responses and Signatures of Metabolic and Epigenetic Dysregulation
Source: Front Cell Infect Microbiol. 2022 Apr 7;12:854242. doi: 10.3389/fcimb.2022.854242 (PMC9067450; doi:10.3389/fcimb.2022.854242)

Supplementary Data

Table 1. Primer sequences for RT-qPCR

Primer sequences were designed using Primer 3. PPIA, GAPDH, PGK1, HRPT1, TBP and HSP90AB1 were used as normalizer genes. All Tm = 60 °C. The relative quantification of the mRNA levels of the target genes was determined using CFX Manager based on the ΔΔCT–method.

Primers	Sequences	Product size (bp)	Accession N° GenBank
Homo sapiens peptidylprolyl isomerase A (PPIA)	GACCCAACACAAATGGTTCCf<br>TCGAGTTGTCCACAGTCAGCr	184	NM_021130.4
Homo sapiens glyceraldehyde-3-phosphate dehydrogenase (GAPDH)	CAAGGGCATCCTGGGCTACf<br>GGTGGTCCAGGGGTCTTACTr	214	NM_002046.5
Homo sapiens phosphoglycerate kinase 1 (PGK1)	GCCACTTGCTGTGCCAAATGf<br>CCCAGGAAGGACTTTACCTTr	102	NM_000291.3
Homo sapiens hypoxanthine phosphoribosyltransferase 1 (HPRT1)	CCCTGGCGTCGTGATTAGTGf<br>TCGAGCAAGACGTTCAGTCCr	139	NM_000194.2
Homo sapiens TATA-box binding protein (TBP),	GCACAGGAGCCAAGAGTGAf<br>GTTGGTGGGTGAGCACAAGr	174	NM_001172085.1
Homo sapiens heat shock protein 90 alpha family class B member 1 (HSP90AB1)	CAGGAACGTACCCTGACTTTGf<br>GCCCAATCATGGAGATGTCGr	145	NM_007355.3
Homo sapiens actinin alpha 2 (ACTN2)	TGGATGCTGAAGACATCGTGAf<br>AAAGCGTGGTAGAAGCAAGAGr	82	NM_001103.3
Homo sapiens absent in melanoma AIM2-001	TGGCAAAACGTCTTCAGGAGf<br>TGCAGCAGGACTCATTTCAGr	101	OTTHUMT00000090341.1
Homo sapiens aldo-keto reductase family 1 member B10 (AKR1B10)	CCAGCACGCATTGTTGAGAAf<br>CAACACGTTACAGGCCCTCCr	108	NM_020299.4
Homo sapiens cadherin 5 (CDH5)	GCAGGCCAGGTATGAGATCGf<br>TGTGTACTTGGTCTGGGTGAAGr	136	NM_001795.4
Homo sapiens cadherin 12 (CDH12)	CAGCATTCTTCAGGGACAACCTTf<br>TCCTCCAAGCTGTCCTCCCATr	145	XM 017008921.1
Homo sapiens cholesterol 25-hydroxylase (CH25H	GGCAACGCAGTATATGAGCGf<br>GAGTGGTCCTCCACGGAAAGr	141	NM_003956.3
Claudin 1	ATGAGGATGGCTGTCATTGGf<br>AGCCTGACCAAATTCGTACCc	147	ENSG00000163347
Homo sapiens colony stimulating factor 2 (CSF2)	CCGGAAACTTCCTGTGCAACf<br>TCCCAGCAGTCAAAGGGGATr	95	NM_000758.3

Homo sapiens complement component 4 binding protein alpha (C4BPA)	TCCCTGGCTACGTCAGATCCf<br>TCCTGGGTGTCTGCATCGTTr	107	NM_000715.3
Homo sapiens C-X-C motif chemokine ligand 6 (CXCL6)	CCTGAAGAACGGGAAGCAAGTf<br>TCCGCTGAAGACTGGGCAATr	144	NM_002993.3
Homo sapiens cytochrome b5 type A (CYB5A)	GGTGACGCTACTGAGAACTTTGf<br>TAAGAGTTTCCGGAGGCTTGr	130	NM_001190807.2
Homo sapiens glutamate ionotropic receptor NMDA type subunit 2A (GRIN2A)	AGACATAGACCCCCTGACCGf<br>AGAACCCCTTGCAGCATTTCr	119	XM_011522461.2
Homo sapiens hydroxycarboxylic acid receptor 2 (HCAR2)	GACAACTATGTGAGGCGTTGGGf<br>CTGCCCTGGCGGTTCATAGr	89	NM_177551.3
Homo sapiens hydroxysteroid 11-beta dehydrogenase 1 (HSD11B1)	TTGGATGGGTTCTTCTCCTCCf<br>CCTTCATGGCTGTTTCTGTGr	109	NM_001206741.1
Homo sapiens hypoxia inducible lipid droplet associated (HILPDA)	GTGGTACTGACCCTACTCTCCAf<br>TGGCTAGTTGGCTTCTGGTGr	109	NM_013332.3
Homo sapiens interleukin 1 beta (IL1B)	ACAGATGAAGTGCTCCTTCCAf<br>GTCGGAGATTCGTAGCTGGATr	73	NM_000576.2
Homo sapiens insulin like growth factor binding protein 3 (IGFBP3)	CCGGGTGTCTGATCCCAAGTf<br>GCGCTGGCTGTCTTTAGCATr	82	NM_000598.4
Homo sapiens interleukin 1 alpha (IL1A)	GTAGCAACCAACGGGAAGGTf<br>AAGGTGCTGACCTAGGCTTGr	127	NM_000575.4
Homo sapiens nicotinamide phosphoribosyltransferase (NAMPT)	CTTCGGTTCTGGTGGAGGTTf<br>TGTTGGGATCAGCAACTGGGr	131	NM_005746.2
NLRP3-001 (NOD-like receptor family, pyrin domain containing 3)	GAAGCACCTGTTGTGCAATCf<br>GCAAGATCCTGACAACATGCr	78	OTTHUMT00000097740.1
Homo sapiens periostin (POSTN)	CCAGCAGTTTTGCCCATTGAf<br>CAGAATAGCGCTGCGTTGTGr	76	NM_001330517.1
Homo sapiens periplakin (PPL)	AGCTGGACAAGCTGAACAACf<br>CCTTGACCTCATTGTGGAAGr	96	NM_002705.4
Homo sapiens phosphoglucomutase 2 like 1 (PGM2L1)	GCAGCCAGAGGCTTGCTAAAf<br>ACCTGCAACTGCTTTGAGCTTr	131	NM_173582.4
Homo sapiens perilipin 2 (PLIN2)	GCCTGTAAGGGGCTAGACAGf<br>CCCCAGTCACAGTAGTCGTCr	130	NM_001122.3
Homo sapiens prostaglandin I2 (prostacyclin) receptor (IP) (PTGIR)	CTCTCACGATCCGCTGCTTCf<br>GGATGGGGTTGAAGGCGTAGr	99	NM_000960.3
Homo sapiens ras homolog family member J (RHOJ)	GCGGGACAGGAGGACTACAA<br>CTTGAGCTCGGGGACCCATT	132	NM_020663.5
Homo sapiens serum amyloid A2 (SAA2)	GCTCGGGGGAACTATGATGCT<br>TCAATGCCTGGGTCATGTAGGAG	139	NM_001127380.2

Homo sapiens superoxide dismutase 2 (SOD2)	GGACAAACCTCAGCCCTAACG<br>TGAGCCTTGGACACCAACAG	132	NM_001322819.2
Homo sapiens sodium voltage-gated channel beta subunit 2 (SCN2B)	CTACCTCGCCCTGCCTTCAG<br>ACTGTGACCTCCATGCTCCG	83	NM_004588.4
Homo sapiens solute carrier family 6 member 15 (SLC6A15)	TTCGCCACATGTTTACCCCT<br>ACACCACCAAATCCCAGACC	106	NM_001146335.2
Homo sapiens suppressor of cytokine signaling 1 (SOCS1)	CGCACTTCCGCACATTCCG<br>CCAGTAGAATCCGCAGGCGT	86	NM_003745.1
Homo sapiens suppressor of cytokine signaling 2 (SOCS2)	TGTGCAAGGATAAGCGGACA<br>GCAGAGATGGTGCTGACGTG	96	XM_017020156
Homo sapiens vanin 1 (VNN1)	TGCCCAATGCCACCCTAACA<br>TATGCGCACCCTGATCTGCT	114	NM_004666.2
Homo sapiens interleukin 32 (IL32)	TGCTTCCCGAAGGTCCTCTCT<br>TGTCCTCAGTGTCACACGCT	118	NM_001308078.3

Table 2. Quality of RNA

RNA quality (RIN) was evaluated using an Agilent 2100 bioanalyzer (Agilent Technologies, Santa Clara, CA). All of the RNA samples had a RIN value greater than 8.2, indicating a good RNA integrity. The ratio 260/280 were greater than 2 indicating a good RNA quality.

Quality metrics							RNA sequencing metrics		
Samples	Biological Replicates	ARN concentration (ng/µl) (nanodrop)	260/280 ratio	RIN	Volume (µl)	Library Quantity (ng)	Trimmed reads total	Mapped Reads	Reads assigned to a gene
Control	Rep1	240.2	2.06	9.0	30	6.1	101,638,072	92,797,996	72,211,182
	Rep2	124.3	2.08	8.2	30	3.7	164,494,388	149,896,316	114,565,856
	Rep3	167.1	2.04	9.1	30	3.9	188,996,872	174,538,262	139,639,291
Positive infected	Rep1	75.61	2.1	8.7	30	2.3	89,692,696	81,848,009	63,146,721
	Rep2	96.62	2.09	9.2	30	2.8	82,726,546	75,085,741	57,480,399
	Rep3	60.1	2.1	8.7	30	2.3	170,519,258	155,464,564	124,294,703

Figure 1. Principal Component Analysis.

PCA indicates that the biological variability (infected vs control) is the main source of variance in the data

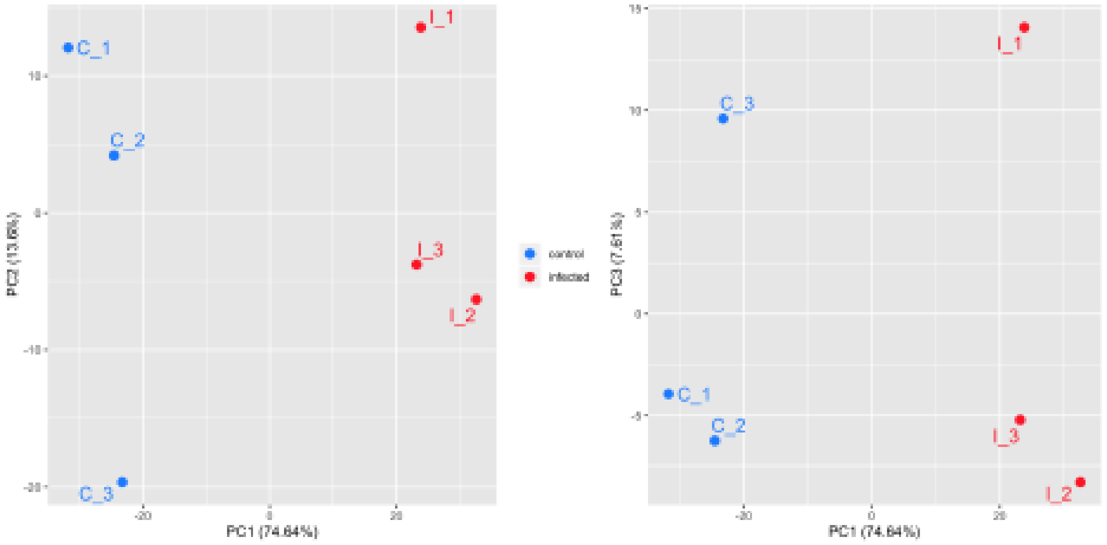

Supplement: Supplementary file 1 [file DataSheet_1.pdf]
